# Human REXO2 controls short mitochondrial RNAs generated by mtRNA processing and decay machinery to prevent accumulation of double-stranded RNA

**DOI:** 10.1093/nar/gkaa302

**Published:** 2020-05-04

**Authors:** Maciej Szewczyk, Deepshikha Malik, Lukasz S Borowski, Sylwia D Czarnomska, Anna V Kotrys, Kamila Klosowska-Kosicka, Marcin Nowotny, Roman J Szczesny

**Affiliations:** 1 Institute of Biochemistry and Biophysics Polish Academy of Sciences, Warsaw 02-106, Poland; 2 Faculty of Biology, Institute of Genetics and Biotechnology, University of Warsaw, Warsaw 02-106, Poland; 3 Laboratory of Protein Structure, International Institute of Molecular and Cell Biology, Warsaw 02-109, Poland

## Abstract

RNA decay is a key element of mitochondrial RNA metabolism. To date, the only well-documented machinery that plays a role in mtRNA decay in humans is the complex of polynucleotide phosphorylase (PNPase) and SUV3 helicase, forming the degradosome. REXO2, a homolog of prokaryotic oligoribonucleases present in humans both in mitochondria and the cytoplasm, was earlier shown to be crucial for maintaining mitochondrial homeostasis, but its function in mitochondria has not been fully elucidated. In the present study, we created a cellular model that enables the clear dissection of mitochondrial and non-mitochondrial functions of human REXO2. We identified a novel mitochondrial short RNA, referred to as ncH2, that massively accumulated upon REXO2 silencing. ncH2 degradation occurred independently of the mitochondrial degradosome, strongly supporting the hypothesis that ncH2 is a primary substrate of REXO2. We also investigated the global impact of REXO2 depletion on mtRNA, revealing the importance of the protein for maintaining low steady-state levels of mitochondrial antisense transcripts and double-stranded RNA. Our detailed biochemical and structural studies provide evidence of sequence specificity of the REXO2 oligoribonuclease. We postulate that REXO2 plays dual roles in human mitochondria, ‘scavenging’ nanoRNAs that are produced by the degradosome and clearing short RNAs that are generated by RNA processing.

## INTRODUCTION

Mitochondria are semiautonomous organelles that possess their own genome. The human mitochondrial genome comprises circular double-stranded DNA that encodes only 37 genes, but each of them is essential. Mitochondrial genes are asymmetrically distributed between mitochondrial DNA (mtDNA) strands, but both mtDNA strands are almost entirely transcribed (1,2). The resulting long polycistronic precursor transcripts are cleaved by RNAse P and ELAC2 protein at tRNA sequences that flank rRNAs and most mRNAs (3,4). Liberated functional RNAs are then post-transcriptionally matured: mRNAs are poly- or oligoadenylated (5), tRNAs are subjected to several nucleotide modifications and the addition of CCA at the 3′ end (6), while rRNAs are methylated and pseudouridylated (7).

The processing of primary mitochondrial RNA (mtRNA) transcripts, especially L-strand-templated precursors, also generates numerous non-coding RNA molecules, the lengths of which range from several dozen to thousands of nucleotides. These RNAs are usually complementary to functional transcripts, raising the possibility of impacting their functionality by hybridization to them. Therefore, steady-state levels of non-coding mtRNAs are controlled and kept very low by mtRNA degradation machinery. The key components of this degradation machinery are SUV3 helicase (8) and polynucleotide phosphorylase (PNPase) (9), which form a functional complex (i.e. the mitochondrial degradosome). Dysfunction of the degradosome-dependent mtRNA decay pathway leads to the accumulation of antisense mtRNAs and further deleterious effects, such as the massive accumulation of double-stranded RNA (dsRNA) that can induce an interferon response (10) or the formation of R loops that interfere with mtDNA maintenance (11). Interestingly, the final products of the mitochondrial degradosome are tetra- or pentanucleotides (12). Short RNAs are also likely to be generated during the processing of primary mtRNA. Thus, another enzyme that is capable of nanoRNA decay must exist in mitochondria.

In *Escherichia coli*, nanoRNAs are degraded by oligoribonuclease Orn, which belongs to DEDD family 3′ to 5′ exonucleases. Orn is a crucial final component of the bacterial mRNA decay pathway and the only essential exonuclease in *E. coli* (13). The human Orn ortholog was proposed to be REXO2, also called small fragment nuclease (Sfn) (14). REXO2 was shown to be active on 5-nucleotide (nt) RNA substrates and possesses a mitochondrial localization signal (14). The functionality of REXO2 was investigated experimentally by Bruni *et al.* (15), who showed that REXO2 was present in both mitochondrial and cytoplasmic compartments. The silencing of REXO2 impaired cell growth and exerted several adverse effects on mitochondrial homeostasis, manifesting as various phenotypes, including mtDNA depletion, the loss of 7S DNA, a decrease in mitochondrial mRNAs, tRNAs and rRNAs, and a decrease in mitochondrial translation levels (15). Thus, REXO2 is important for proper mitochondrial gene expression and cell survival. However, unknown is whether these phenotypes depend on the ribonucleolytic activity of REXO2 in mitochondria. Physiological mitochondrial REXO2 substrates have also not been identified.

Here, we report comprehensive functional, biochemical, and structural studies to elucidate REXO2 function in human mitochondria. Using a cellular model developed by us, we demonstrate that loss of the catalytic activity of REXO2 in mitochondria resulted in the accumulation of diverse non-coding mtRNA species. This population included short, linear RNAs that are primary substrates of the enzyme, such as ncH2 RNA, which we describe here for the first time. The population also included longer, structured molecules, including tRNA-like, that cannot be degraded by REXO2 on its own, implying that the removal of short RNAs by REXO2 is required for the proper function of other RNA-degrading entities (i.e. the mitochondrial degradosome). We show that the accumulation of REXO2-controlled RNAs affected the mitochondrial degradosome, leading to the upregulation of mitochondrial dsRNA. We also demonstrate that REXO2 degrades RNA in a structure- and sequence-dependent manner.

## MATERIALS AND METHODS

### Cell culture and the development of stable cell lines

Most of the experiments were performed using HeLa Flp-In T-REx cells (gift from Matthias Hentze) (16) or their stably transfected derivatives that were generated in this study. In the REXO2 immunolocalization experiments, we also used cell lines that were obtained from the American Type Culture Collection (ATCC; A549 [ATCC CCL-185], BT-474 [ATCC HTB-20] and MCF10A [ATCC CRL-10317]) or were a kind gift from Johannes Spelbrink (143B) and Jacek Jaworski (human primary fibroblasts). All of the cells were cultured at 37°C under a 5% CO_2_ atmosphere in Dulbecco's modified Eagle's medium (DMEM; Gibco) that was supplemented with 10% fetal bovine serum (Gibco), with the exception of MCF10A cells, which were cultured in dedicated medium that was recommended by ATCC. The expression of miRNA and transgenes was induced with 100 ng/ml tetracycline. The identity of HeLa and 143B cells was confirmed by DSMZ (Germany). Stable cell lines were established using protocols that were described in detail elsewhere (17). The plasmids that were used to generate stable cell lines are listed in Supplementary Table S1.

### Immunofluorescence

For localization studies of endogenous REXO2, cells were plated on glass coverslips and cultured for 24 h, followed by immunostaining that was performed according to a previously described procedure (18), with the exception that incubation with primary antibody (rabbit polyclonal anti-REXO2, Abcam, ab206694) was performed for 2 h at room temperature instead of overnight incubation at 4°C. For transgene-expressing cell lines, the cells were plated on coverslips in medium that contained tetracycline and cultured for 72 h, followed by REXO2 immunostaining (described above) or dsRNA immunostaining with J2 primary antibodies according to a previously described procedure (10).

### Subcellular fractionation

The expression of exogenous variants of REXO2 was induced for 3 days with tetracycline (100 ng/ml) in HeLa stable cell lines, and cells from two 145 mm dishes were trypsinized. The cells were then centrifuged at 400 × *g* for 4 min at 4°C. The cell pellet was suspended in 5 ml of cold NKM buffer (1 mM Tris–HCl [pH 7.4], 130 mM NaCl, 5 mM KCl and 7.5 mM MgCl_2_), and 180 μl was taken as the total fraction. The cells were centrifuged at 400 × *g* for 4 min at 4°C. The cell pellet was suspended in 1 ml of 0.1× HomB buffer (4 mM Tris–HCl [pH 7.6], 2.5 mM NaCl and 0.5 mM MgCl_2_) and incubated on ice for 5 min. The cells were homogenized in 2 ml Dounce homogenizer, and then 10× concentrated HomB buffer (400 mM Tris–HCl [pH 7.6], 250 mM NaCl and 50 mM MgCl_2_) was added to obtain a 1× concentration. The cell homogenate was transferred to a 1.5 ml tube and centrifuged three times at 900 × g for 3 min at 4°C. For each centrifugation, the supernatant was transferred to a new tube. The mitochondria were then pelleted by centrifugation at 10 000 × *g* for 10 min at 4°C. Protease inhibitors were added to the supernatant, which was frozen at −20°C and saved as the cytoplasmic fraction. Mitochondria were suspended in 1 ml of 1× HomB and centrifuged at 10 000 × *g* for 10 min at 4°C. Mitochondrial and untreated cell pellets (total fraction) were lysed in 150 μl of 1× HomB with the addition of protease inhibitors and 1% Triton X-100. Lysates were stored at −20°C before subjecting them to SDS-PAGE. Subcellular fractions were subjected to SDS-PAGE on a 12% gel. Proteins were subjected to wet transfer to an Amersham Protran nitrocellulose membrane (Merck) and stained with Ponceau S. Membranes were blocked in 5% nonfat milk in TBS for 1 h at room temperature. The following primary antibodies were used: rabbit polyclonal anti-REXO2 (Abcam, ab206694, 1:5000 dilution), mouse monoclonal anti-LRPPRC (Santa Cruz Biotechnology, sc-166178, 1:4000 dilution), and mouse monoclonal anti-α-tubulin (Merck, CP06, 1:10 000 dilution). Membranes were incubated with primary antibodies in 5% nonfat milk overnight at 4°C. Membranes were washed with TBS and then incubated with appropriate horseradish peroxidase-conjugated secondary antibodies (Calbiochem, 401393 or 401215, 1:10 000 dilution) in 5% nonfat milk for 1 h at room temperature. Membranes were washed with TBS, and antibodies were visualized using ECL kit (BioRad).

### Analysis of mt-dsRNA stability

Cells were cultured on a 384-well plate for 72 h in the presence of tetracycline (100 ng/ml). Mitochondrial transcription was inhibited by actinomycin D (Sigma, A1410) at a final concentration of 5 μg/ml for the indicated time. The cells were then washed twice with phosphate-buffered saline (PBS), fixed in PBS that contained 4% formaldehyde (Sigma), 0.25% Triton X-100 (Sigma) and Hoechst 33342 (2 μg/ml, Molecular Probes), washed three times with PBS, and blocked in 3% bovine serum albumin (BSA) (Sigma) dissolved in PBS. The cells were incubated overnight at 4°C with J2 anti-dsRNA primary antibodies (English and Scientific Consulting Kft, 10010500) at a concentration of 2.5 μg/ml and anti-AIF antibodies (0.4 μg/ml; Santa Cruz Biotechnology, sc-13116). The cells were washed three times with PBS and incubated for 1 h at room temperature with appropriate secondary antibodies that were conjugated to AlexaFluor dyes. The cells were washed three times with PBS and left in PBS. A Multidrop Combi Reagent Dispenser (Thermo Fisher Scientific) was used for cell plating and the addition of all solutions to the 384-well plate. A 405 LS Microplate Washer (BioTek) was used for the washing steps. Cell imaging was performed using a ScanR automated microscope (Olympus) with a UPlanSApo 20.0× objective. Image analysis was performed using ScanR 2.7.2 software (Olympus). The signal from nuclear DNA stained with Hoechst dye was used to identify individual cells. The spot detector module was used to identify the objects representing the dsRNA. In addition, dsRNA objects were gated to those located only in mitochondria by using a fluorescent signal from anti-AIF antibodies.

### Measurement of mtDNA transcription

mtDNA transcription was measured using a previously described procedure (19). The analysis was performed on 384-well plates. Cells were plated with a Multidrop Combi Reagent Dispenser (Thermo Fisher Scientific). Transgene expression was induced with tetracycline (100 ng/ml) for 72 h. Bromouridine (BrU; 2.5 mM, Sigma) was added to the cell culture medium for 30 min. After incubation with BrU, the cells were washed twice with PBS and fixed in 4% formaldehyde (Sigma) with 0.25% Triton X-100 (Sigma) and Hoechst 33342 (2 μg/ml, Molecular Probes) in PBS. The cells were then washed three times with PBS and blocked in 3% BSA (Sigma). The cells were incubated overnight at 4°C with anti-BrdU (BrU) primary antibodies (0.5 μg/ml, Santa Cruz Biotechnology, sc-32323) and anti-AIF (0.4 μg/ml; Santa Cruz Biotechnology, sc-13116). The cells were then washed three times with PBS and incubated for 1 h at room temperature with appropriate secondary antibodies that were conjugated to AlexaFluor dyes. The cells were then washed three times with PBS and left in PBS. All of the solutions were added using a Multidrop Combi Reagent Dispenser (Thermo Fisher Scientific). The washing steps were performed using a 405 LS Microplate Washer (BioTek). Cell imaging was performed with a ScanR fluorescence microscope system that was adapted for high-throughput image acquisition (Olympus) using a UPlanSApo 20.0× objective. Images were analyzed using ScanR 2.7.2 analysis software (Olympus). The signal from nuclear DNA stained with Hoechst dye was used to identify individual cells. The spot detector module was used to identify the objects representing newly synthesized RNAs. In addition, newly synthesized RNA objects were gated to those located only in mitochondria by using a fluorescent signal from anti-AIF antibodies.

### Western blot

Total protein cell extracts were prepared in RIPA lysis solution that was supplemented with 1% (v/v) Triton X-100 and 1% (w/v) deoxycholate. Protein concentrations were measured using the Bradford method, and 30 μg protein from each extract was separated by SDS-PAGE and transferred to a PVDF membrane (Protran). Western blot was performed using standard protocols with rabbit polyclonal anti-REXO2 primary antibody (Abcam, ab206694, 1:2000 dilution) and horseradish peroxidase-conjugated secondary antibody (Calbiochem, 401393, 1:10 000 dilution), which were detected using a ECL kit (BioRad, 170-5061).

### RNA isolation and northern blots

Both total RNA and mitochondrial RNA were isolated from cells that were cultured for 6 days in the presence of an miRNA/transgene expression inducer. Total RNA was isolated using TRI reagent (Sigma) according to the manufacturer's instructions, resolved on 1% denaturing agarose gels, and analyzed using a standard northern blot procedure as described previously (8) with [α-32P] UTP-labeled strand-specific riboprobes (Supplementary Table S2). The level of tRNA-like transcripts was analyzed as described previously (18). A different procedure was used to analyze short mtRNAs. We isolated mitochondria from cells from six confluent 145 mm plates per sample using a previously described method (8). The final mitochondrial pellets were either frozen in liquid nitrogen and stored at −80°C or directly used for small RNA isolation using the mirVana miRNA Isolation Kit (Ambion, AM1560). We applied the manufacturer's protocol to allow the enrichment of small (<200 nt) RNA fractions. One modification of this procedure was the second re-extraction of RNA from the organic phase, which was previously shown to significantly increase the RNA yield (20). Next, 200 ng of the small mtRNA fraction was denatured in formamide loading dye for 3 min at 85°C, run on a 15% polyacrylamide/8 M urea/1× TBE gel in a Mini-Protean II apparatus (BioRad), visualized with GelRed stain (Biotium), and electrotransferred to Hybond-NX membranes (GE Healthcare) in 0.5× TBE buffer for 16 h at a constant current of 20 mA using a Mini Trans-Blot Cell apparatus (BioRad). RNA was chemically cross-linked to membranes with l-ethyl-3-(3-dimethylaminopropyl) carbodiimide (EDC) as previously described (21). Small mitochondrial transcripts were detected using the following PNK-radiolabeled complementary oligoprobes: RSZ943 CTTCAAACCTGCCGGGGCTTCTCCCGCCTTTTTT (OriL primer), RSZ944 CCCGGCGGCGGGAGAAG (nc-OL), and RSZ946 GGGGGTGTCTTT (ncH2). Hybridizations were performed overnight at 37°C in PerfectHyb Plus buffer (Sigma), followed by three washes with 2× SSC solution for 10 min at 37°C. The membranes were then exposed to PhosphorImager screens (FujiFilm). The results were recorded in a Typhoon FLA 9000 scanner (GE Healthcare). Finally, the data were analyzed using Multi Gauge 3.0 software (FujiFilm). For high-resolution northern blots, 3 μg of total RNA was denatured as described above, resolved in 15% denaturing sequencing polyacrylamide gel for 3 h at a constant power of 20 W, and electrotransferred to Hybond-NX membranes in 0.5× TBE buffer for 16 h at a constant voltage of 10 V. Following chemical cross-linking, hybridization was performed as described above.

### Library preparation and next-generation sequencing

The RNA-seq experiments were performed in three or two biological replicates per cell line. Isolated RNA was purified from DNA contaminations using TURBO DNase (Ambion, AM2238), followed by phenol-chloroform extraction and ethanol precipitation. Ribosomal RNA was depleted using the Ribo-Zero rRNA Removal Kit (Illumina). We prepared strand-specific dUTP libraries according to a previously described protocol (22), with minor modifications that were described previously (17). Library quality was verified using a Bioanalyzer 2100 apparatus (Agilent). High-throughput sequencing was performed using an Illumina NextSeq 500 platform with the NextSeq 500 High Output Kit (Illumina) and standard pair-end sequencing procedures (2 × 75 cycles).

### Bioinformatic analysis of next-generation sequencing data

The raw RNA-seq data were trimmed of adapters using cutadapt 1.9.2.dev0 software. Low-quality bases and improperly paired reads were removed prior to mapping using Trimmomatic 0.32 software. Trimmed reads were mapped to the human genome (GRCh38) using the STAR 2.5.2b aligner and basic GENECODE v26 annotation with introduced features that allowed the quantification of all reads that were mapped in the D-loop region. Mapped reads were counted using htseq-count 0.9.1 software. Differential expression analysis was performed using the edgeR 3.18.1 package. Wiggle files of uniquely mapped reads were converted to the BigWig format using of wigToBigWig package. Tracks of mapped reads were visualized using Integrative Genomics Viewer and assembled using CorelDraw software. The contribution of L-strand RNAs to the mitochondrial transcriptome was calculated as described previously (18).

### Heterologous overexpression and protein purification

N-terminal-truncated REXO2 (Δ28aa) was expressed in *E. coli* BL21(DE3) RIL cells as N-terminal 6× His-SUMO-tagged fusion protein. Bacteria that were transformed using specific expression vectors (Supplementary Table S1) were grown in LB medium at 37°C until an optical density at 600 nm (OD_600_) of 0.6–0.7 was reached, and then expression was induced by the addition of IPTG to a final concentration of 1 mM. Bacteria were then cultured for an additional 16 h at 18°C. Bacterial cells were pelleted by centrifugation at 4000 × *g* for 20 min at 4°C, resuspended in lysis buffer (50 mM NaH_2_PO_4_, 300 mM NaCl, 10 mM imidazole and protease inhibitor cocktail [Roche]), and lysed by sonication. Lysates were centrifuged at 16 000 × *g* for 30 min at 4°C. The supernatants then underwent the following steps: Ni^2+^ affinity chromatography on Ni-NTASuperflow resin (Qiagen), SUMO protease on-column tag cleavage, desalting and a second round of Ni^2+^ affinity chromatography, in which the cleaved 6× His-SUMO tags were retained on the column, and unbound material that contained REXO2 was collected. Finally, a Hiload 16/60 Superdex 200 column (GE Healthcare) was used for gel filtration. The entire procedure was performed using an ÄKTA express apparatus (GE Healthcare).

For crystallization studies, a similar purification approach was applied, with several modifications. Cells were induced with 0.04 mM IPTG and grown overnight at 16°C. Cells were lysed by sonication in buffer that contained 50 mM Tris–HCl (pH 7.5), 2 M NaCl, 40 mM imidazole, 5% glycerol and 5 mM 2-mercaptoethanol. A HisTrap column (GE Healthcare) was used for affinity purification. A Superdex 200 size exclusion column (GE Healthcare) was equilibrated with 20 mM HEPES (pH 7.5), 100 mM NaCl, 0.5 mM Tris(2-carboxyethyl) phosphine hydrochloride (buffer B), and 5 mM (CH3COO)_2_Ca or (for long-term protein storage) buffer B supplemented with 10% glycerol. SUV3 and PNPase were purified as described previously (18).

### Crystallization

HPLC-purified RNA oligonucleotides were purchased from Metabion. Protein and RNA were mixed in a 1:1 ratio before crystallization. Single-stranded RNA oligonucleotides, ranging in length from 7 to 11 nt, were used for co-crystallization (Supplementary Table S3). All of the crystallization experiments were performed at 18°C using the sitting drop vapor diffusion method. Initial hits were obtained using Index Screen (Hampton Research). Diffracting crystals were obtained in 0.2 M potassium sodium tartrate trihydrate and 20% PEG3350. Crystals were obtained after mixing an equal volume of protein (12.5 mg/ml) and reservoir solution. For data collection, crystals were flash frozen in liquid nitrogen, and 30% glycerol was used as the cryoprotectant.

### Diffraction, data collection, structure solution and refinement

X-ray diffraction data were collected at PETRA III Hamburg at a 0.89440 Å wavelength. Data processing was performed using XDS (23). The statistics for diffraction data are summarized in Supplementary Table S4. Molecular replacement was used to solve the structure in the Phenix Phaser module (24). The model that was used for molecular replacement was oligoribonuclease from *Acinetobacter baumnii* (PDB ID: 5CY4, Seattle Structure Genomics Centre for Infectious Diseases), which shares 48.3% sequence identity with REXO2. Two dimers were obtained after structure solution. COOT (25) was used for manual model building. Refinement was performed using Phenix (24). The analysis of the reflection file in Xtriage from Phenix showed the presence of twinning in the diffraction data. The twinning law -h,-k, l was applied during the refinement process. After several rounds of refinement, the electron density for the RNA molecule in one of the active sites was less well defined.

### RNA degradation assays

Oligonucleotide substrates either were radiolabeled with [γ-32P] ATP (Hartmann Analytic) using T4 PNK (NEB) or carried a 5′ fluorescein modification. Radiolabeled oligonucleotides were subjected to phenol–chloroform extraction, precipitated with ethanol, and purified by electrophoresis in 15% denaturing polyacrylamide gels. All of the oligonucleotide substrates that were used in the present study are listed in Supplementary Table S3. REXO2 activity assays were performed at 37°C in a 20–40 μl reaction volume that contained 20 mM Tris–Cl (pH 7.4), 50 mM NaCl, 2.5 mM MnCl_2_, 0.1 mM DTT, 50 nM REXO2 and 500 nM substrate. If applicable, differences in the reaction conditions are noted in the figure legends. Reactions were stopped at the indicated time points by the addition of an equal volume of RNA loading dye (95% formamide, 25 mM EDTA [pH 8.0], 0.01% [w/v] xylene cyanol, and 0.01% [w/v] bromophenol blue), followed by freezing in liquid nitrogen. Reaction products were denatured for 5 min at 85°C and analyzed on 15% or 20% polyacrylamide denaturing gels (8 M urea, 1× TBE). The gels were exposed for 6–48 h (depending on signal strength) to PhosphorImager screens (FujiFilm), which were then processed with a Typhoon FLA 9000 scanner (GE Healthcare). The results were quantified and analyzed using Multi Gauge 3.0 software (FujiFilm). To study the effects of nanoRNA removal by REXO2 on mitochondrial degradosome activity, we used purified PNPase and SUV3 preparations prepared as described previously (18). Reactions were performed in a 30 μl volume that contained 50 mM NaCl, 10 mM Tris–Cl [pH 7.5], 1 mM Na_2_HPO_4_, 1 mM MgCl_2_, 1 mM MnCl_2_, 1 mM DTT, 1 mM ATP, 150 nM PNPase, 50 nM SUV3, 100 nM REXO2, 50 nM 5′-FAM-tRNA-like oligonucleotide, and 5 μM unlabeled 5RNA oligonucleotide. All of the other procedures were identical to the procedures that are described above for the REXO2 activity assays.

## RESULTS

### Establishment of a cellular model to study mitochondrial functions of REXO2

**Figure 1.**
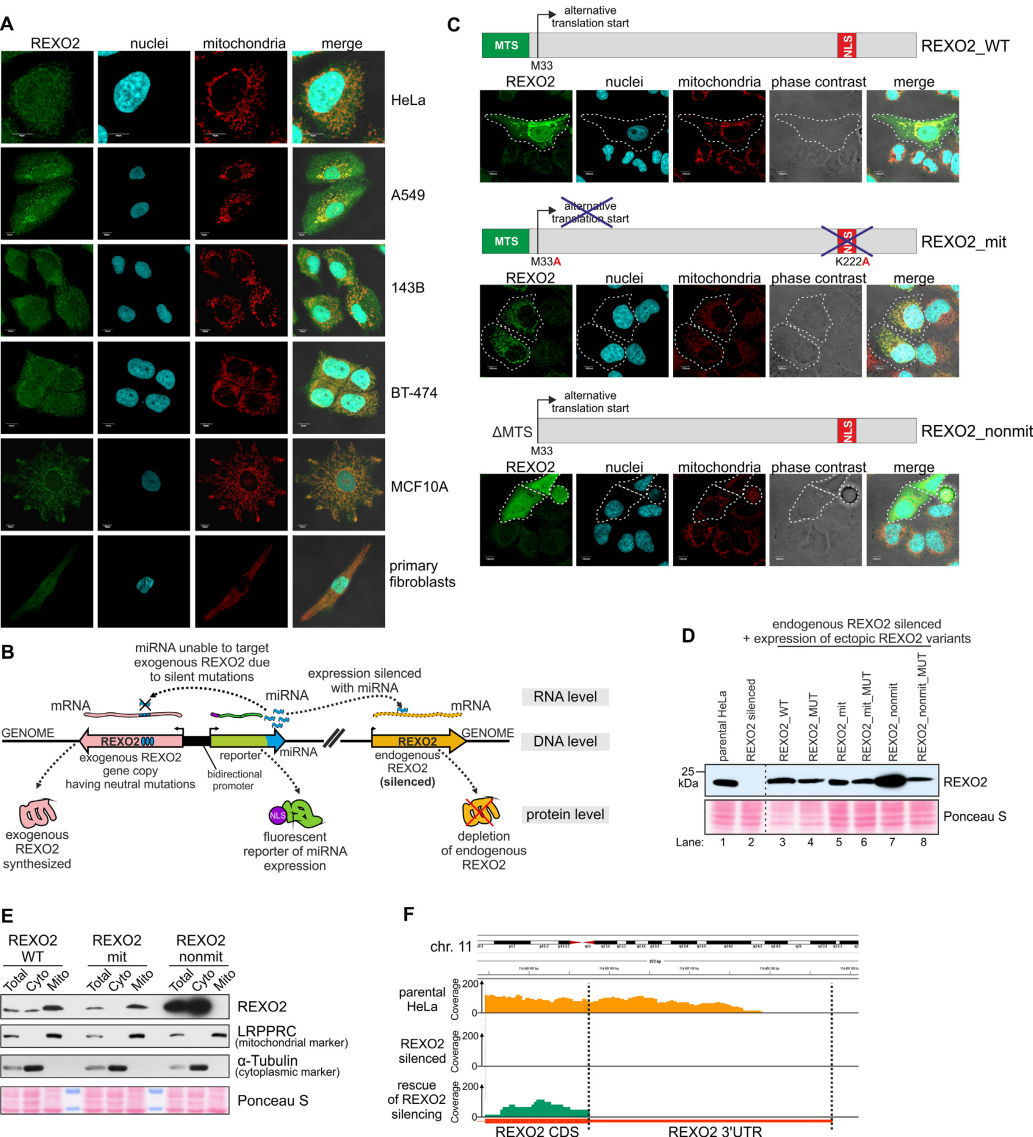
The established cellular model allows functional studies of REXO2 in mitochondria. (**A**) Intracellular localization of endogenous REXO2 in different cell lines by confocal microscopy. Immunofluorescence was performed using anti-REXO2 antibodies. Nuclei and mitochondria were stained with Hoechst and MitoTracker CMXRos Red, respectively. Scale bars represent 10μm. (**B**) Schematic model of the engineered cell lines. (**C**) Schematic representation of ectopic REXO2 variants and their localization verified by transient transfection followed by anti-REXO2 immunofluorescence. Dashed lines indicate transfected cells. REXO2_WT, full-length wild-type protein; REXO2_mit, protein localized only to mitochondria; REXO2_nonmit, truncated REXO2 that lacked the mitochondrial targeting sequence. (**D**) Western blot analysis of REXO2 levels in engineered cell lines. Anti-REXO2 antibodies were used. PonceauS staining was used as a loading control. (**E**) Western blot analysis of exogenous variants of REXO2 in total, cytoplasmic, and mitochondrial fractions. LRPPRC and α-tubulin were used as markers of mitochondria and the cytoplasm, respectively. PonceauS staining was used as a loading control. (**F**) Analysis of REXO2 silencing efficacy at the RNA level. Tracks of RNA-seq reads mapped to the REXO2 gene. Parental HeLa cells (parental HeLa), cells with silenced REXO2 (REXO2 silenced), and cells that expressed ectopic REXO2_WT that replaced the miRNA-silenced endogenous copy of REXO2 (rescue of REXO2 silencing) are shown.

Previous studies (14,15) indicated that REXO2 can be expressed as two isoforms: a full-length mitochondrial precursor and a truncated variant that starts from methionine at position 33 (M33), which lacks the mitochondrial targeting signal (MTS). Cell fractionation studies confirmed that REXO2 is localized to both the cytoplasm and mitochondria (15). Additionally, it was suggested that REXO2 may possess a nuclear localization signal (NLS) (14), which is consistent with the results of our *in silico* analysis (in which a NucPred (26) score of 0.55 was found for the truncated REXO2 variant) but this was not previously verified experimentally. Thus, we performed immunofluorescence studies of the intracellular localization of REXO2 in several cell lines of diverse tissue origin (Figure 1A) using antibodies, the specificity of which was validated using human HeLa cells with silenced or overexpressed REXO2 (Supplementary Figure S1). We confirmed previous observations that REXO2 is primarily a mitochondrial protein since REXO2 staining was co-localized with mitochondria in all of the tested cell lines (Figure 1A). Nevertheless, we also detected REXO2 in the cytoplasm and cell nuclei. Therefore, to study the direct role of REXO2 in mtRNA metabolism, we developed a cellular model that allows discernment of the mitochondrial and non-mitochondrial functions of REXO2. Such a model should also allow examinations of whether mtRNA phenotypes of REXO2 depletion are linked to nucleolytic activity of the protein. To obtain the model, we applied a strategy previously described by us (17,27), which enables inducible, miRNA-directed depletion of the protein of interest. In addition, this approach allows miRNA silencing of the endogenous copy of the gene of interest and the simultaneous expression of its ectopic, miRNA-insensitive version. As a result, the endogenous version of the studied protein is replaced by its exogenous form (Figure 1B). Additionally, miRNAs are co-transcriptionally expressed with a fluorescent reporter that enables the tracking of miRNA-expressing cells.

We designed three variants of ectopic REXO2 alleles that encoded various forms of REXO2 protein (Figure 1C) that could serve as positive and negative controls: (i) a full-length protein (REXO2_WT), (ii) a mitochondrial-only variant with mutations in predicted nuclear localization signal and alternative translation start site (REXO2_mit), (iii) a truncated non-mitochondrial variant lacking N-terminal 32 residues that form mitochondria-targeting sequence (REXO2_nonmit; Figure 1C). Analyses of intracellular localization of the transgenes that were transiently expressed in HeLa cells showed their expected localization (Figure 1C). The full-length protein (REXO2_WT) was present in mitochondria, nuclei, and the cytoplasm. The mitochondrial version (REXO2_mit) was observed only in these organelles (Figure 1C). The non-mitochondrial form of REXO2 (REXO2_nonmit) was not targeted to mitochondria (Figure 1C). To study the role of ribonucleolytic activity of REXO2, catalytically inactive versions of all three variants were also created, in which aspartate 168 was changed to alanine because this mutation was previously shown to render the protein inactive (14).

We established stable HeLa cell lines that inducibly expressed REXO2-targeting miRNAs alone (Figure 1D, lane 2) or miRNAs and the aforementioned ectopic REXO2 variants (Figure 1D, lanes 3–8). In most cell lines, exogenous REXO2 was expressed at nearly physiological levels (Figure 1D) and had the predicted intracellular localization, which was verified by cellular fractionation (Figure 1E). The silencing of endogenous REXO2 was very efficient: REXO2 bands were not observed on the Western blot (Figure 1D), and reads that mapped to the REXO2 locus were not observed when RNA from the respective cell line was analyzed by RNA-seq (Figure 1F). Additionally, analysis of the RNA-seq data from the cell line that expressed ectopic REXO2_WT revealed reads that mapped to the coding sequence of REXO2 but not to the 3′ untranslated region (Figure 1F), confirming that the signal that was observed on the western blot (Figure 1D) represented only exogenous protein because it was expressed from a gene that includes only the coding sequence. These findings confirmed that the established model was suitable for functional studies.

### REXO2 depletion in mitochondria leads to the accumulation of non-coding transcripts that originate from transcription of the L-strand of mtDNA

The aforementioned experiments validated our established cellular model, which was then used to examine the global effect of REXO2 dysfunction on the mitochondrial transcriptome. We performed RNA-seq experiments using RNA that was isolated from cells with silenced REXO2 expression, and as controls: parental HeLa cells and cells that expressed miRNA-insensitive full-length REXO2_WT (rescue). We applied strand-specific library preparation protocol described by us before (17).

**Figure 2.**
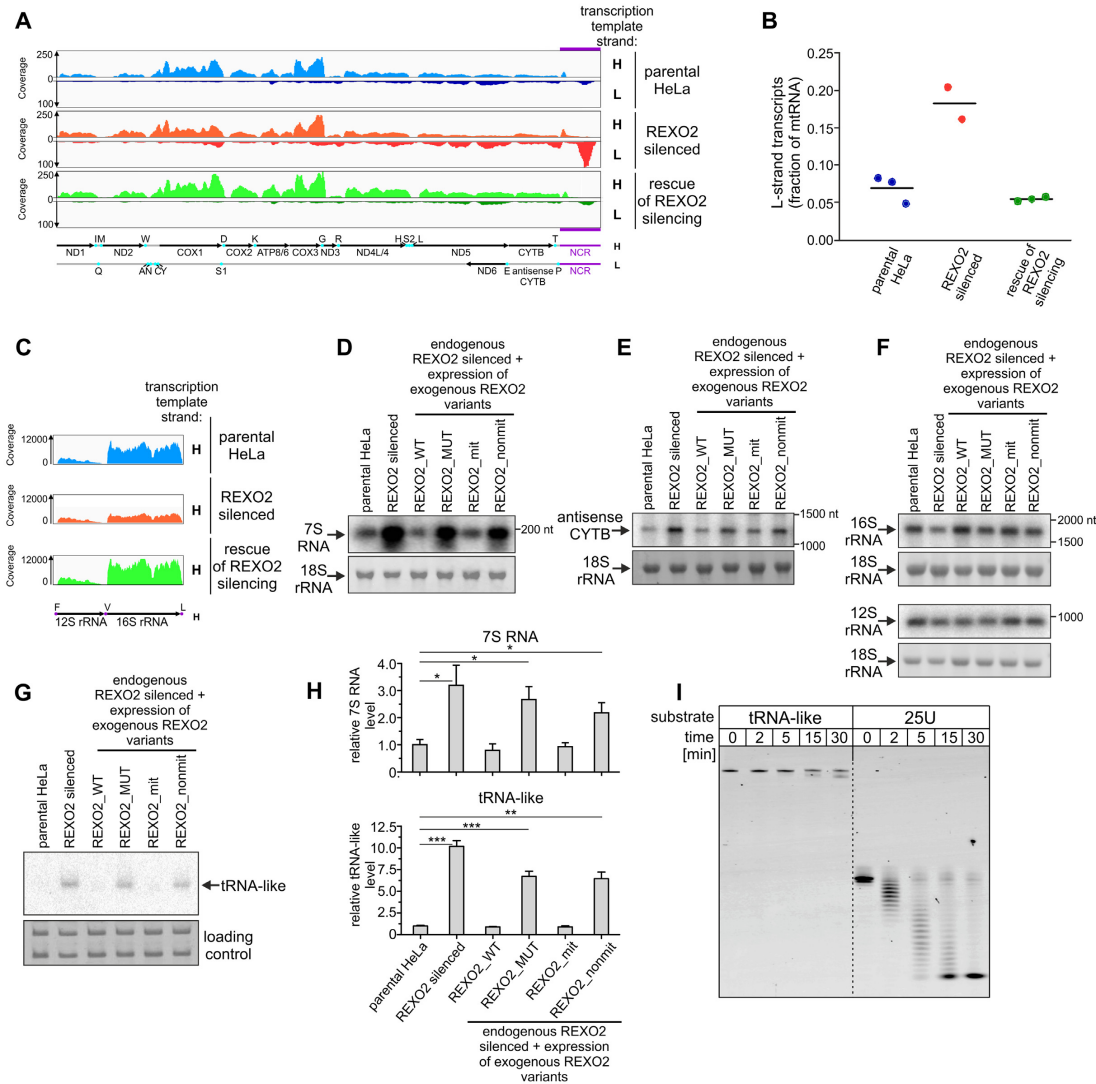
Depletion of REXO2 results in the accumulation of antisense L-strand-templated transcripts. (**A**, **C**) Tracks of RNA-seq reads mapped to the mitochondrial genome. RNA isolated from parental HeLa cells, REXO2-silenced cells, and cells that expressed ectopic REXO2_WT that replaced miRNA-silenced endogenous REXO2 (rescue of REXO2 silencing) was analyzed. The mitochondrial rRNA region is shown separately in C because of different coverage. The experiment was performed in triplicate. A map of mitochondrial transcripts is shown. The NCR region is highlighted in purple. (**B**) Graph of the contribution of L-strand-templated transcripts to the mitochondrial transcriptome. Individual values are shown. Horizontal lines represent mean values. (**D**–**F**) Confirmation of RNA-seq results by northern blot hybridization to detect 7S RNA, antisense CYTB, and mtDNA-encoded rRNAs, respectively. Nuclear-encoded 18S rRNA (methylene blue staining) was the loading control. (**G**) Northern blot analysis of tRNA-like. Nuclear-encoded 5.8S and 5S rRNAs (methylene blue staining) were the loading controls. (**H**) Quantification of northern blot analysis of tRNA-like and 7S RNA levels (mean ± SEM from three biological replicates) calculated relative to parental HeLa cells. A two-tailed unpaired *t*-test was applied (***P <0.001, **P 0.001 to 0.01, *P 0.01 to 0.05). (**I**) Results of *in vitro* degradation assay with the indicated substrates and recombinant REXO2.

Mapping of the sequenced reads to the mitochondrial genome did not reveal significant changes in the levels of protein-coding transcripts (Figure 2A). On the other hand, we observed a global increase in levels of non-coding RNAs that mostly originate from transcription of the L-strand. Consequently, we observed a three-fold increase in the contribution of L-strand-templated RNAs to the mitochondrial transcriptome (Figure 2B). Notably, the distribution of mtDNA-mapped reads indicated that the non-coding region (NCR) was the most affected by REXO2 depletion, as we observed more than a 10-fold increase in the number of reads that mapped to this region (Figure 2A). Additionally, we detected more than a two-fold decrease in 16S rRNA upon REXO2 silencing. This change was limited only to 16S rRNA; the 12S rRNA transcript was unaffected (Figure 2C). Importantly, all of the observed phenotypes were fully rescued by the expression of an ectopic gene that encoded catalytically active REXO2 (Figure 2A–C).

To confirm the RNA-seq data, we performed northern blot analyses using strand-specific riboprobes that were complementary to two L-strand transcripts: 7S RNA (encoded in the NCR region) and antisense of CYTB mRNA. We also analyzed the levels of both mitochondrial rRNAs that are products of H-strand transcription. Apart from the samples that were used in the RNA-seq experiments, we also included RNA that was isolated from additional control cell lines that expressed the REXO2 variants that are described above (i.e. REXO2_MUT, REXO2_mit, and REXO2_nonmit). The northern blot results were consistent with the RNA-seq results. Upon the silencing of REXO2, we observed the massive accumulation of 7S RNA (Figure 2D), a significant increase in antisense CYTB (Figure 2E), and a decrease in 16S rRNA without apparent changes in 12S rRNA levels (Figure 2F). All of the phenotypes were rescued only when nucleolytically active mitochondrially localized REXO2 variants were expressed (REXO2_WT and REXO2_mit) (Figure 2D-F), indicating that the observed changes in mtRNAs resulted from lack of the nucleolytic activity of REXO2 in mitochondria and were not secondary effects of functional impairments of REXO2 in non-mitochondrial compartments.

The observed global increase in L-strand transcripts upon REXO2 silencing resembled phenotypes that were reported by us previously when mitochondrial degradosome function was disrupted (18). Therefore, we analyzed whether the impairment of REXO2 function in mitochondria affected tRNA-like levels, which are regulated by the degradosome (18). We found that tRNA-like levels increased more than 10-fold upon REXO2 silencing, a phenotype that was rescued by the expression of catalytically active REXO2 that possessed MTS but not its inactive or MTS-deleted variants (Figure 2G, H).

To determine whether tRNA-like is a physiological substrate of REXO2, we tested the ability of the enzyme to degrade such a structured substrate *in vitro*. REXO2 was heterologously expressed in *E. coli* and affinity-purified, resulting in a high-purity preparation of the enzyme (Supplementary Figure S2A). The preliminary activity assay confirmed that the purified REXO2 degraded short (5 nt) single-stranded RNA (Supplementary Figure S2B). Next, we tested REXO2 activity toward a synthetic oligoribonucleotide whose sequence corresponded to tRNA-like. As a control, we used a 25 nt poly(U) homopolymer (i.e. a substrate that is significantly longer than the substrates that were used previously in REXO2 activity studies (14,28) but was predicted to be devoid of any secondary structure). REXO2 efficiently degraded the linear (U)_25_ substrate, but it was unable to completely degrade tRNA-like, only trimming 2–3 nucleotides from the 3′ end was observed (Figure 2I). Thus, tRNA-like is unlikely to be a physiological REXO2 substrate, and its accumulation is likely an indirect, possibly degradosome-dependent phenotype.

### REXO2 silencing exerts diverse effects on small mtRNAs, including the massive accumulation of a novel short RNA species

**Figure 3.**
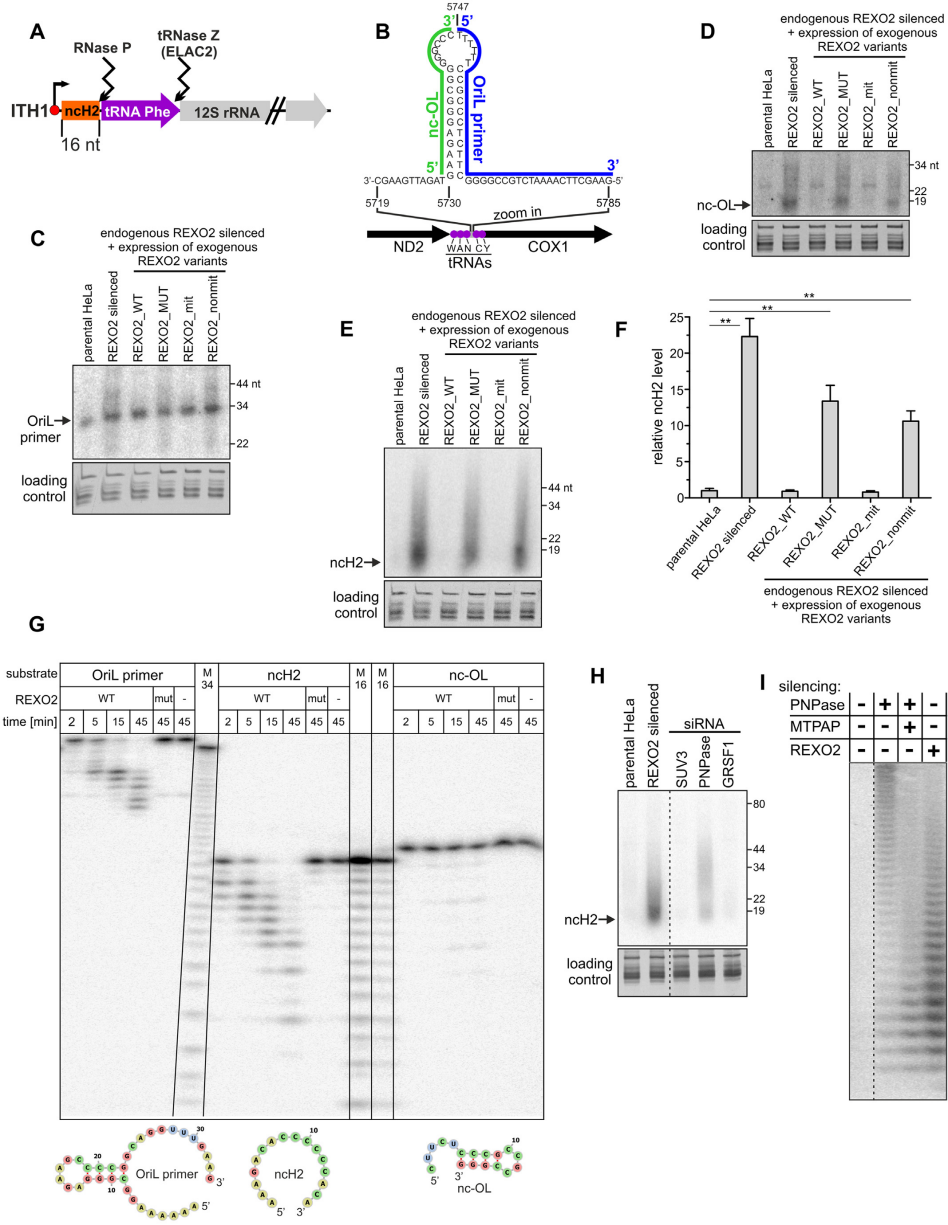
REXO2 silencing results in the upregulation of novel short RNA species. (A, B) Schematic representations of short transcripts. (**A**) Transcription initiation site (ITH) and the excision of tRNA lead to the formation of ncH2. (**B**) For nc-OL and the OriL primer, the OriL stem-loop region is shown. (**C**-**E**) Northern blot analysis of OriL primer, nc-OL transcript, and ncH2 transcript, respectively. Blots were performed on samples that were enriched with short (< 200 nt) mitochondrial RNAs. tRNA pool (GelRed staining) was the loading control. (**F**) Quantification of northern blot analysis of ncH2 levels (mean ± SEM, three biological replicates) calculated relative to parental HeLa cells. A two-tailed unpaired *t*-test was applied (**P 0.001 to 0.01). (**G**) Results of RNA degradation assay with recombinant REXO2 and the indicated substrates, whose sequences correspond to transcripts that were detected by northern blots that are shown in C-E. Secondary structures of the studied RNAs that were predicted by RNAfold software are shown in the bottom panel. WT, wild-type REXO2; mut, REXO2 mutant (D47A D168A); M34 and M16, RNA size markers obtained by alkaline hydrolysis. (**H**) Northern blot analysis of ncH2 levels in REXO2, mitochondrial degradosome subunits (SUV3 and PNPase), and GRSF1-silenced cells. Total cellular RNA samples were analyzed. (**I**) High-resolution northern blot analysis of ncH2 upon the silencing or co-silencing of the indicated genes. Total cellular RNA samples were analyzed.

Available data indicated that very short RNA molecules are primary substrates of REXO2 (14,15,28). Such RNAs can arise as end- or by-products of the decay and processing of longer transcripts. However, knowledge of the existence of such RNAs in mitochondria is limited. Sequencing of the mitochondrial transcriptome (29) identified several short (20–40 nt) species, mostly tRNA-derived fragments, that are unlikely to be directly controlled by REXO2 because this enzyme is unable to degrade structured RNAs (Figure 2I). The identification of very short transcripts by RNA-seq poses a technical problem because they are difficult to be unequivocally mapped to the genome, and they are prone to be lost during RNA isolation. Thus, we searched for potential *in vivo* substrates of REXO2 by *in silico* analysis of the distribution of processing sites in primary mtRNA and positions of transcription start sites. This allowed us to identify at least two short (<20 nt) RNAs that could theoretically be produced by mtDNA transcription and the processing of RNA precursors. To our knowledge, however, their existence has not been previously verified experimentally. The first RNA, which we named ncH2, is a 16-nt molecule that should arise from the initiation of transcription at the ITH1 site, followed by tRNAPhe excision (Figure 3A). The second predicted RNA was a 17-nt RNA that originated from the transcription of mtDNA upstream of the locus where the OriL transcript was mapped (Figure 3B). We named this putative transcript nc-OL.

For both of the predicted short mtRNAs (ncH2 and nc-OL), we examined whether their levels are regulated by REXO2. We also analyzed the OriL transcript, a known mitochondrial short RNA that serves as a primer for mtDNA replication (29,30). The silencing of REXO2 resulted in the appearance of RNA species that were both longer and shorter than the main OriL primer band, but no significant quantitative changes were observed for the latter (Figure 3C). In contrast, the levels of the remaining two transcripts increased upon REXO2 silencing (Figure 3D, E), especially ncH2, which showed massive ∼22-fold increase in accumulation (Figure 3E, F). Importantly, the presence of catalytically active REXO2 in mitochondria that was caused by the expression of REXO2_WT or REXO2_mit transgenes rescued the aforementioned phenotypes, indicating that these changes were caused by dysfunction of the mitochondrial fraction of REXO2 (Figure 3C–F). To examine whether the accumulation of ncH2 upon REXO2 silencing depended on mtDNA transcription, we treated REXO2-silenced cells with the transcription inhibitor actinomycin D and measured steady-state levels of ncH2. No significant changes were observed after actinomycin D treatment, indicating that ncH2 upregulation in REXO2-silenced cells did not depend on mtDNA transcription (Supplementary Figure S3).

To determine which of the RNAs that were investigated above are a direct REXO2 substrate, we performed *in vitro* RNA degradation assays. While synthetic ncH2 was degraded by REXO2, other examined substrates were either almost fully resistant (nc-OL) or only partially degraded (OriL primer) by the enzyme (Figure 3G). This difference in susceptibility of the substrates to REXO2-mediated degradation was consistent with their ability to form secondary structures. RNA fold predictions showed that ncH2 was linear, whereas nc-OL was almost fully double-stranded, and the OriL primer formed a stem-loop with single-stranded tails at the 5′ and 3′ ends (Figure 3G). Notably, a pattern of OriL degradation suggested that REXO2 degraded only the 3′ single-stranded tail of this substrate. Altogether, these biochemical experiments indicated that ncH2 is a direct substrate of REXO2, which is consistent with the finding that among the analyzed transcripts, ncH2 accumulated to the greatest extent upon REXO2 silencing.

### ncH2 accumulation affects degradosome function

Next, we examined whether the accumulation of REXO2 substrates (e.g. ncH2) upon dysfunction of this enzyme leads to impairments of degradosome function. We assumed that the substrate should meet specific criteria to affect degradosome function. It should constitute the *in vivo* substrate of SUV3 and/or PNPase and be highly abundant to divert these enzymes from their other substrates. To address the former criterion, we silenced the degradosome components SUV3 and PNPase as well as GRSF1, which we showed to cooperate with the degradosome in mtRNA surveillance (18). We then examined ncH2 levels by northern blot. We found ncH2 accumulation in PNPase-depleted cells but not in SUV3- or GRSF1-silenced cells (Figure 3H). This suggests that accumulated ncH2 can sequester one subunit of the degradosome (PNPase). Notably, ncH2 degradation by PNPase did not appear to require SUV3 or GRSF1, which was unsurprising because ncH2 is unstructured. Thus, it can be fully degraded by PNPase alone since previous studies showed that PNPase degrades linear RNA without the assistance of other factors (31). Next, we estimated the amount of ncH2 in the cell by the northern blot analysis of total cellular RNA that was spiked with various amounts of synthetic ncH2 (Supplementary Figure S4). This analysis revealed that in REXO2-silenced cells, ncH2 existed in ∼600 000 copies per cell, which was 10-fold higher than the most abundant mt-mRNA in HeLa cells (32), thus classifying ncH2 as a highly abundant transcript. These results are consistent with the hypothesis that ncH2 upregulation that is caused by the depletion of REXO2 can impact degradosome activity by ‘sequestering’ its ribonucleolytic subunit.

Interestingly, the ncH2-specific oligoprobe did not detect a single band but rather a smear above the predicted ncH2 size, indicating the heterogeneous length of the transcript (Figure 3E, H). This may have resulted from the initiation of transcription at variable sites or posttranscriptional modification (i.e. polyadenylation of the 3′ end). Additionally, ncH2 species that accumulated upon PNPase silencing seemed to be longer than when REXO2 was depleted (Figure 3H). Indeed, nucleotide-resolution analysis using PAGE confirmed that ncH2 species that were present in PNPase-depleted cells were longer (Figure 3I).

To see whether heterogeneity of the length of ncH2 resulted from its non-templated adenylation, we treated RNA samples with RNA-DNA-specific RNAse H in the presence of exogenously added synthetic oligo-dT and then analyzed reaction products by gel electrophoresis. This analysis confirmed our presumption that ncH2 is adenylated (Supplementary Figure S5). Moreover, we found that this modification was mediated by mitochondrial poly(A) polymerase (MTPAP) because the *in vivo* co-silencing of this enzyme in PNPase-depleted cells decreased the length of ncH2 (Figure 3I).

Overall, these findings indicated that highly abundant ncH2 is a direct substrate of REXO2, the accumulation of which may affect other mtRNA transactions, including degradosome-mediated mtRNA decay. We also found that the adenylation status of ncH2 impacted its decay pathway, with REXO2 preferentially controlling non-adenylated or poorly adenylated species.

### REXO2 is required for the maintenance of proper mt-dsRNA levels

Our previous work demonstrated that expression of the mitochondrial genome is a source of cellular dsRNA in humans (10). The key players that are responsible for controlling mt-dsRNA levels are degradosome components, SUV3 and PNPase. Impairments of either SUV3 or PNPase leads to the massive accumulation of mt-dsRNA (10). Many of the aforementioned REXO2 silencing phenotypes mirrored effects of degradosome dysfunction, suggesting that the clearance of short RNAs by REXO2 is an important step to sustain proper degradosome function. If so, we hypothesized that REXO2 depletion should affect the levels of mt-dsRNA.

**Figure 4.**
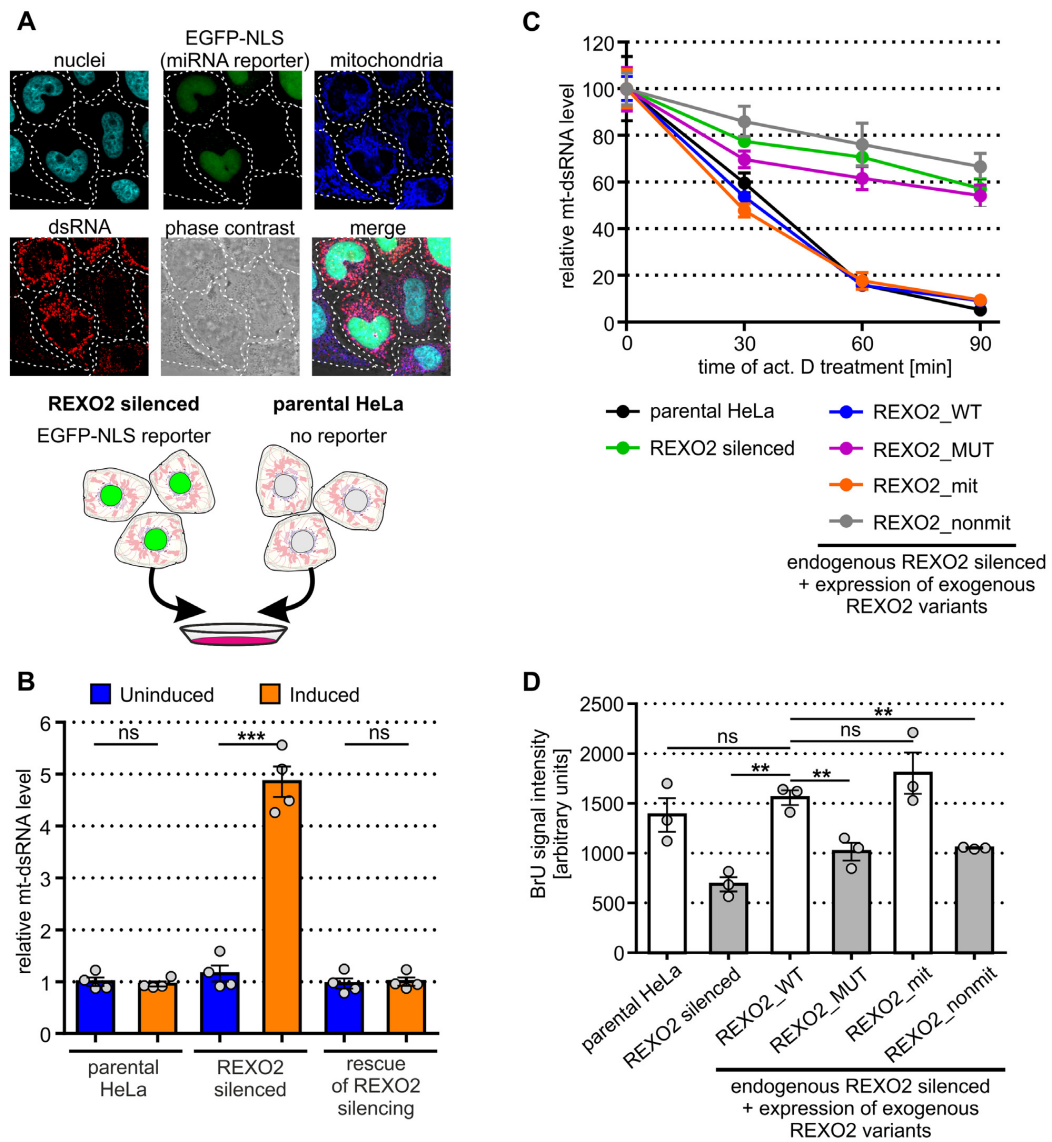
REXO2 silencing results in the accumulation of dsRNA in mitochondria. (**A**) Immunofluorescence analysis of dsRNA levels in parental and REXO2-depleted HeLa cells. A schematic representation of the experimental design is shown in the bottom part of the panel. dsRNA-specific J2 antibodies were used. Nuclei and mitochondria were stained with Hoechst and MitoTracker DeepRed, respectively. (**B**) Quantitative analysis of mt-dsRNA levels in the studied cell lines using automated fluorescence microscopy. Measurements were performed 3 days after miRNA and transgene expression induction. Bars show mean ± SEM of total mt-dsRNA foci intensity values calculated from tetraplicates, where in each replicate >1200 cells were analyzed. Mean of each replicate is shown as a dot. (**C**) Analysis of mt-dsRNA stability. The indicated cells were treated (or not) with actinomycin D, and mt-dsRNA levels were measured using J2 immunostaining. Mean (±SEM) of total intensity values of mt-dsRNA foci are shown. Four replicates were analyzed (each replicate > 500 cells). (**D**) Measurement of mtDNA transcription. The indicated cells were cultured in the presence of BrU and the amount of BrU that was incorporated into mitochondrially localized RNAs was quantified by anti-BrU immunostaining and fluorescent microscopy. Bars show the mean of the replicates ± SEM. Dots represent means of replicates (> 650 cells in each replicate). A two-tailed unpaired *t*-test was applied (***P < 0.001, **P 0.001 to 0.01, ns – not significant P > 0.05).

To test this hypothesis, we analyzed mt-dsRNA in control and REXO2-depleted cells by subjecting them to immunofluorescence staining using dsRNA-specific monoclonal J2 antibodies. Our previous studies confirmed the specificity of J2 for dsRNA (10). For direct comparisons of control and REXO2-depleted cells, we utilized our established cellular model, in which the expression of REXO2-targeting miRNA was coupled with expression of the fluorescent reporter, thus allowing the recognition of miRNA-positive cells (i.e., REXO2-depleted cells). We mixed two types of cells: (i) cells that co-expressed miRNAs that targeted REXO2 and the EGFP-NLS reporter, (ii) parental cells that lacked the miRNA cassette and fluorescent reporter (Figure 4A). The respective mixture was plated and subsequently immunostained on the same coverslip, allowing side-by-side comparisons of REXO2-silenced and control cells. We observed a strong increase in the dsRNA signal that co-localized with mitochondria in cells with silenced REXO2, indicating that REXO2 contributed to the regulation of mt-dsRNA (Figure 4A, cells expressing EGFP in nuclei). Importantly, this result was validated by a rescue experiment, in which we analyzed the levels of mt-dsRNA in cells that expressed miRNA-insensitive REXO2 (REXO2_WT). We found that the expression of exogenous REXO2_WT restored physiological levels of mt-dsRNA in cells that were depleted of endogenous REXO2 (Figure 4B).

Dysfunction of the degradosome upon REXO2 silencing should result in the stabilization of mt-dsRNA, an effect that was previously observed when SUV3 or PNPase was silenced (10). Therefore, we measured the stability of mt-dsRNA and found that depletion of the mitochondrial fraction of REXO2 led to the stabilization of mt-dsRNA (Figure 4C). The upregulation of mt-dsRNA upon REXO2 depletion could also have resulted from an increase in mtDNA transcription. Therefore, we examined transcription of mtDNA using labeling of newly synthetized mtRNAs with bromouridine (BrU). We cultured cells in the presence of BrU and then we analyzed the levels of mitochondrially localized BrU-labelled transcript with the help of immunostaining and fluorescent microscopy (19). We did not observe an increase in mtDNA transcription in REXO2-silenced cells. In fact, a reduction of mtRNA synthesis was observed in cells that were depleted of the mitochondrial fraction of REXO2 (Figure 4D).

Altogether, these data indicate that function played by REXO2 is required to prevent dsRNA accumulation in mitochondria. They also suggest that the effect on mt-dsRNA is attributable to a decrease in mtRNA degradation and not an increase in mitochondrial transcription.

### REXO2 substrate specificity reveals its putative direct substrates

**Figure 5.**
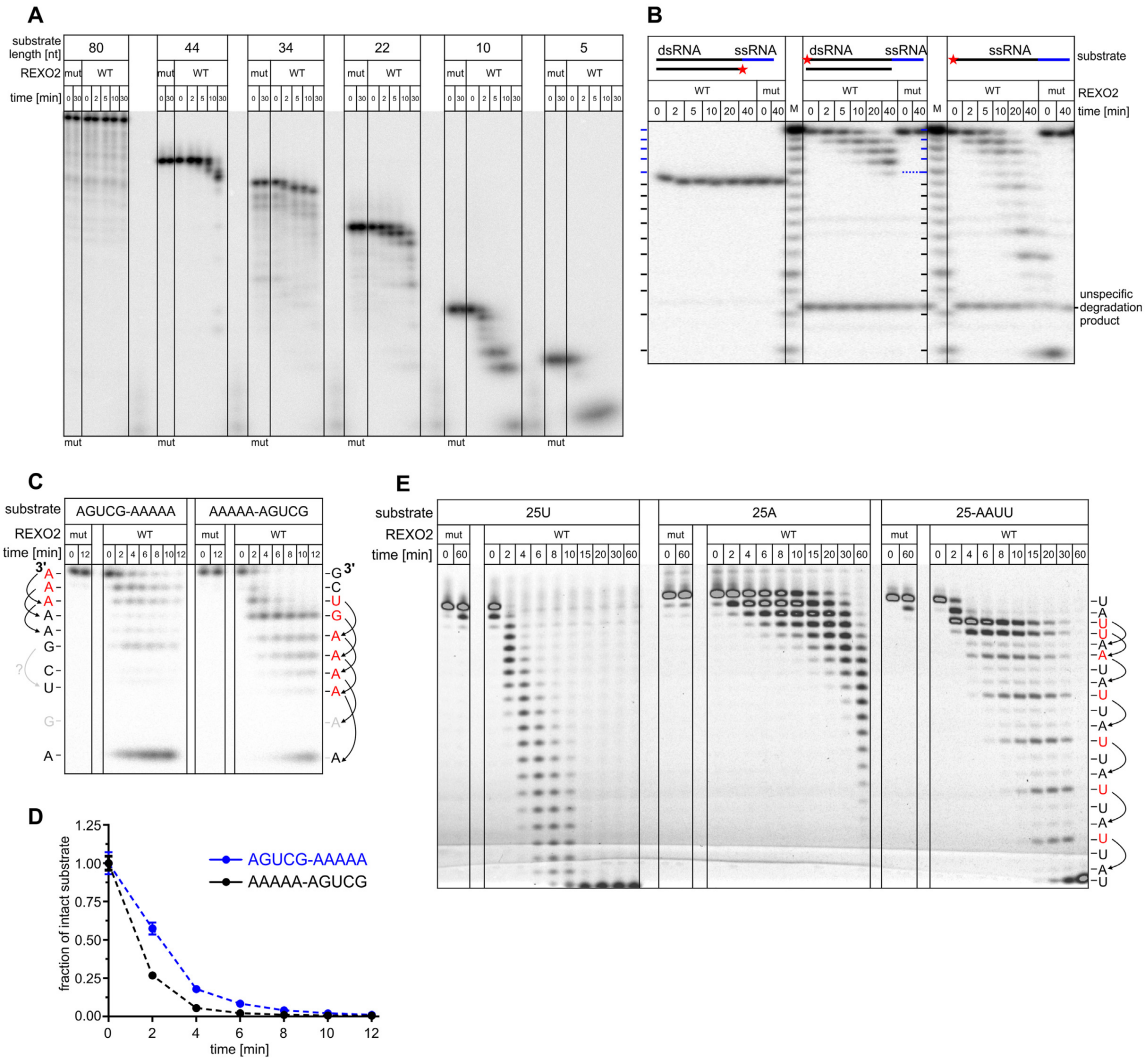
REXO2 substrate specificity. (**A–C**, **E**) *In vitro* degradation of the indicated substrates by indicated REXO2 recombinant variants. WT, wild-type REXO2; mut, REXO2 mutein (D47A D168A); M, RNA size markers obtained by alkaline hydrolysis. In B, the dsRNA substrate was obtained by annealing 10RNA (CGACUGGAGC) with RNA10comp-5A (GCUCCAGUCGAAAAA). dsRNA has 5 adenines as a single-stranded overhang (marked in blue). The asterisk indicates the 5′ radiolabeled strand. In sequence specificity studies (C, E), the 3′-end nucleotide of degradation by-products of AGUCG-A_5_, A_5_-AGUCG, and 25-AAUU substrates is indicated. Degradation slowdowns are marked in red, and upstream bases that are putatively responsible for each slowdown are marked by arrows. Substrates in A-C were radiolabeled. In E, 5′ fluorescein-labeled oligonucleotides were used. (**D**) Quantification of substrates degradation that are shown in C. The data are expressed as the mean ± SEM of three independent experiments.

The above experiments identified several transcripts with diverse lengths and structures that are affected by depletion of the mitochondrial REXO2 pool. To further explore which of these transcripts can be direct substrates of REXO2, we performed *in vitro* studies of REXO2 substrate specificity. We first investigated the effect of substrate length on REXO2 activity. REXO2 trimmed RNAs as long as 44 nt, but degradation efficiency was inversely proportional to RNA length, and the longest oligonucleotide (80 nt) was not cleaved (Figure 5A). Next, since the results of the *in vivo* experiments indicated the involvement of REXO2 in controlling dsRNA levels we studied whether this nuclease is able to degrade dsRNA substrates. We annealed two partially complementary oligonucleotides, forming a short dsRNA substrate with a 3′ single-stranded overhang (Figure 5B). REXO2 degraded the 3′ overhang, but the double-stranded part of the substrate was intact (Figure 5B). This was consistent with *in vitro* degradation of the other structured substrates, including the tRNA-like, nc-OL and OriL transcripts (Figures 2G, 3G). Importantly, the control reaction showed that REXO2 fully degraded the applied radiolabeled substrate when it was not in duplex with a complementary oligonucleotide (Figure 5B).

Subsequently, we studied the effect of RNA adenylation on RNA degradation by REXO2. The *in vivo* experiments with ncH2 suggested that adenylation status of the substrate modulates its susceptibility to degradation by REXO2 (Figure 3H, I). To study this issue, we used two 10-nt substrates that had the same nucleotide composition but a different sequence order, with the (A)_5_ tract positioned either on the 5′ or 3′ end (Figure 5C). The quantification of decay of the full-length substrates suggested that homopoly(A) tracts were not the preferred REXO2 substrates (Figure 5D). Interestingly, the degradation patterns had some peculiarities. Cleavage of the three 3′-end adenines was visibly slow with the 3′-adenylated substrate; the remaining adenines from this 5-nt tract were cleaved much faster (Figure 5C, D). Moreover, for the 5′ adenylated oligonucleotide, degradation slowed not after reaching the first adenine but rather earlier, i.e., 2 nt ahead of the adenine tract (Figure 5C). This implied that REXO2 activity may be influenced by the identity of a base 2 nt upstream of the 3′ end. Sequence analysis suggested that the poorest nucleotide cleavage occurred when the third position from the 3′ end was occupied by an adenine (Figure 5C). To explore this further, we performed an additional assay using three 25-nt-long substrates: poly(A) and poly(U) homopolymers and the oligonucleotide RNA25-AAUU that was composed of 64% uridines and 36% adenines. All of the substrates were predicted to be unstructured. Consistent with the previous results, the poly(A) substrate was the slowest to be degraded (Figure 5E). Moreover, for the heterogeneous substrate, we detected slower degradation when adenine was the third nucleotide from the 3′ terminus (Figure 5E). Altogether, these findings indicated that neither long (>40 nt) mtRNAs nor mt-dsRNA/structured mtRNAs are direct substrates of REXO2. Moreover, these results revealed that REXO2 shows sequence preference.

### REXO2 is a homodimer with two equivalent catalytic centers that accommodate single-stranded RNA

**Figure 6.**
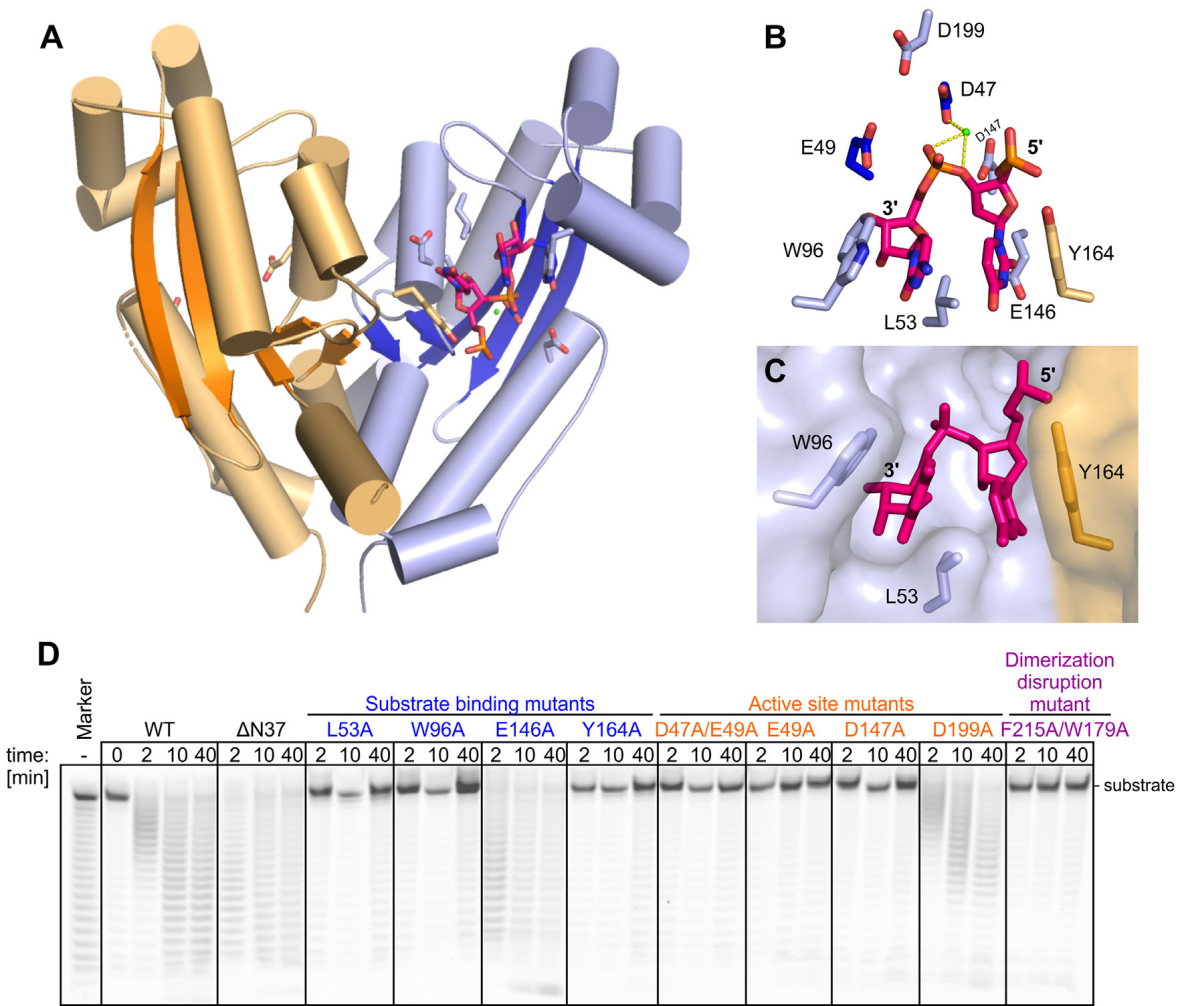
Crystal structure of REXO2 (28–226) and verification of residues involved in catalytic activity. (**A**) The two protomers are in orange and blue with β-strands in darker shades of the same color. The RNA molecule, active site residues, and residues that are involved in RNA binding are shown as sticks. The Ca^2+^ ion is shown as a green sphere. (**B**) Magnification of the active site and residues that are involved in RNA binding. Tyr164 from the other protomer of the dimer is shown in orange. (**C**) Aromatic clamp showing the first two nucleotides from the 3′ end positioned between W96 from one molecule and Y164 from the other molecule. The protein is shown in surface representation, and two nucleotides of the RNA and selected protein residues are shown as sticks. (**D**) Poly(U)25 digestion by different mutants of REXO2.

To understand the molecular basis of the biochemical properties of REXO2, we attempted to solve its crystal structure. We crystallized the full-length protein that lacked the N-terminal MTS sequence. These crystals diffracted X-rays to 3.1 Å resolution and belonged to the *P* 3_2_ space group. The structure was solved by molecular replacement using oligoribonuclease (ORN) from *Acinetobacter baumnii* (PDB ID: 5CY4) as a search model and refined to an *R*_free_ of 22.17%. The asymmetric unit of the crystal contained two dimers of REXO2. The protomers of the dimer adopted a typical RNase H fold of the DEDDh superfamily with a central five-stranded β-sheet with strands that were ordered 3-2-1-4-5 and strand 2 that ran antiparallel to the others (Figure 6A) (33). REXO2 is structurally similar to other members of the DEDDh superfamily. For example, REXO2 could be superimposed on closely related ORN with a root-mean-square deviation (rmsd) of 1.0 Å (164 C-α) with essentially the same positions of all secondary structure elements. The dimerization of REXO2 mostly involved the helix located after strand 4, strand 5, and the helix located after strand 5.

The active site comprised Asp47, Glu49, Asp147, Asp199 and His194 and had the typical architecture of the DEDDh superfamily (Figure 6B). The sidechains of carboxylate residues normally coordinate two divalent metal ions. We observed only one Ca^2+^ ion that was coordinated by Asp47 (Figure 6B). The presence of only one ion is most likely attributable to the fact that Ca^2+^ ions do not support catalysis. Each dimer in the asymmetric unit interacted with one molecule of RNA that was located at the active site (Figure 6A). Both RNA molecules comprised three nucleotides with an AUC sequence. The 3′ terminus of the RNA was located in a pocket that was formed by Glu49, His101, Cys97 and the backbone of Met50 and was shielded from the solvent. This means that the RNA could not continue in the 3′ direction; therefore, the active site could not accommodate a continuous RNA chain. This explains why REXO2 is an exonuclease. Additionally, the base of the 3′-terminal nucleotide formed a stacking interaction with Trp96. The base of the adjacent nucleotide formed a stacking interaction with Tyr164 from the other protomer of the dimer. Trp96 and Tyr164 formed a clamp, holding the two 3′-terminal nucleotides that were linked by the phosphate group that was hydrolyzed by REXO2. Leu53 was inserted between the two bases, further stabilizing binding by the aromatic clamp. The base edges of the two 3′-terminal RNA residues formed few interactions (Figure 6C). Glu146 was located in the vicinity of the 5′ base and may have formed steric clashes with larger purine rings. High B-factors of the third nucleotide indicated that it was highly mobile. Thus, two 3′-terminal nucleotides of the RNA formed the minimal substrate of REXO2.

To verify the importance of the functional residues that were identified in the structure, we prepared REXO2 variants with substitutions of these residues and tested their activity on poly(U) substrates. These variants could be grouped into three categories. The first category comprised substitutions at the active site (E49A, D147A, D199A and D47A/E49A). These variants were completely inactive, with the exception of D199A, which played an auxiliary role in catalysis (Figure 6D). The second category comprised variants with substitutions in residues that were involved in substrate binding (L53A, W96A, E146A and Y164A). In agreement with the structure, substitutions in the aromatic clamp (W96A and Y164A) and the stabilizing residue Leu53 led to the complete absence of activity (Figure 6D). E146A was as active as WT on the poly(U) substrate, indicating that this residue did not play a major role in substrate binding (Figure 6D). The third category was REXO2 with an alanine substitution of two residues that were involved in dimerization (Phe215 and Trp179). We used gel filtration coupled with multi-angle light scattering and found that this variant was monomeric, in contrast to WT protein that formed a dimer (Supplementary Figure S6). This dimerization-deficient variant was completely inactive (Figure 6D). This is in agreement with the structure, which showed that both subunits of the dimer provided residues to formation of the aromatic clamp.

In the crystal packing, we observed an interesting interaction of the N-terminal helix of REXO2 from one protomer of one dimer with another dimer in the crystal lattice (Supplementary Figure S7). To determine whether this interaction is functionally important, we prepared a variant of REXO2 with the deletion of this helix (Δ37N). This mutant retained full activity (Figure 6D), indicating that the interaction that was mediated by the N-terminal helix was a crystallization artifact.

### nanoRNA removal by REXO2 supports the degradation of longer structured substrates by the mitochondrial degradosome

In the above experiments, we found that REXO2 degraded longer RNAs than those that were reported previously (14,15,28). Nevertheless, its substrate specificity was restricted to single-stranded, unstructured nucleic acids, suggesting that the accumulation of RNAs with strong secondary structures or dsRNA upon REXO2 silencing was an indirect effect of REXO2 dysfunction. Furthermore, some phenotypes of REXO2 silencing, such as the global increase in mitochondrial non-coding L-strand transcripts, resembled the known effects of degradosome dysfunction. Thus, we hypothesized that the removal of short RNAs that are primary REXO2 substrates is required for proper function of the degradosome complex. We tested this hypothesis using *in vitro* degradation assays.

**Figure 7.**
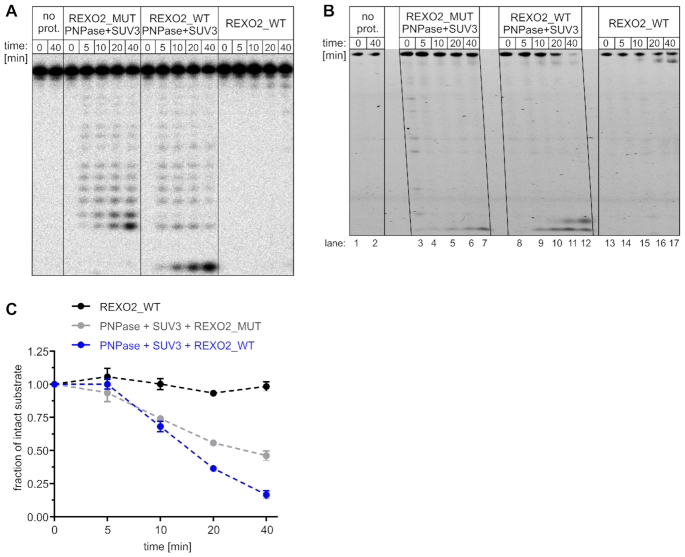
REXO2 degrades nanoRNAs that may impair degradosome activity. (**A**) *In vitro* degradation of radiolabeled 17-nt-long, partially structured substrate by recombinant REXO2, mitochondrial degradosome (SUV3 and PNPase), and their combined action. REXO2_WT, wild-type REXO2; mut, catalytically inactive REXO2 (D47A D168A); no prot., no protein control. (**B**) *In vitro* degradation of tRNA-like oligonucleotide by the indicated proteins (abbreviations as in A) in the presence of an excess of nanoRNA (5RNA). (**C**) Quantification of tRNA-like decay that is shown in B. The data are expressed as the mean ± SEM of three independent experiments.

We first investigated whether end products of degradosome activity were recycled to mononucleotides by REXO2. As a substrate, we used RNA-17, an oligonucleotide that is predicted to possess a hairpin structure and thus is protected from degradation by REXO2 itself but degraded by the degradosome. To allow the visualization of all degradation by-products, we used 10-fold lower enzyme concentrations relative to the other assays. The shortest products of degradosome activity were tetranucleotides, which were degraded to mononucleotides in the presence of catalytically active REXO2 (Figure 7A). Importantly, full-length RNA-17 was not degraded by REXO2 alone (Figure 7A). Thus, REXO2 ‘scavenged’ nanoRNAs produced by the SUV3–PNPase complex.

To confirm that nanoRNA accumulation leads to impairments of degradosome function, we used a different approach. We tested combined activity of REXO2 and degradosome using a mixture of two substrates: tRNA-like and nanoRNA (5RNA). The latter, REXO2-targeted substrate was present in excess in the reaction to mimic the accumulation of nanoRNAs upon REXO2 silencing. As expected, tRNA-like was only slightly trimmed by REXO2 alone (Figure 7B, lanes 13–17) and degraded by the degradosome (Figure 7B, C). However, the efficiency of tRNA-like degradation by the SUV3-PNPase complex was visibly higher when catalytically active REXO2 was present in the reaction mixture (Figure 7B, C). Importantly, REXO2 did not augment the degradation of tRNA-like in the absence of excess 5RNA (Supplementary Figure S8), which would be expected if REXO2 is required for the decay of short RNA (5RNA) but not tRNA-like. Altogether, these findings indicate that REXO2 is a factor that cleans nanoRNAs, which in turn prevents impairments of degradosome-mediated RNA decay.

## DISCUSSION

RNA decay is a key element of RNA metabolism, necessary for maintaining proper mRNA levels, elimination of aberrant transcripts and processing by-products, and recycling of monoribonucleotides that are required for transcription. Such nucleases as REXO2 are key players in RNA decay. While the first work identifying REXO2 as a homologue of bacterial oligoribonuclease was published 20 years ago and it already suggested dual nuclear/mitochondrial subcellular localization of the protein (14), REXO2 functions in eukaryotic cells remained largely unknown. REXO2 silencing was reported to have deleterious effects on human mitochondria, resulting in their disorganized network of punctate and granular structure and a decrease in mitochondrial nucleic acid content (15). However, because no rescue experiments were performed, it remained unclear whether the observed phenotypes were directly related to REXO2 oligoribonuclease activity in mitochondria. Surprisingly, the affected transcripts were mostly mRNAs, i.e. long transcripts that are unlikely to be regulated directly by an oligoribonuclease. While the present study was under review Nicholls *et al.* reported that REXO2 knockout in mice resulted in early embryonic death, thus demonstrating the essential function of REXO2 (34). However, conditional tissue-specific knockout did not significantly alter the steady-state levels of mtRNA (34), thus leaving unanswered questions about the function of REXO2 in mtRNA surveillance.

In the present study, we developed a cellular model that enabled discernment of the mitochondrial and non-mitochondrial functions of REXO2 and verification of whether the observed phenotypes are linked to nucleolytic activity of the protein. Using engineered cell lines, we demonstrated, for the first time, the importance of REXO2 in controlling the levels of mitochondrial antisense transcripts and dsRNA. We also identified at least one physiological substrate of REXO2 other than nanoRNAs, namely ncH2, a novel and abundant short transcript that was produced by mtRNA processing machinery.

We found that ncH2 degradation occurred independently of the mitochondrial degradosome complex because SUV3 helicase depletion had no effect on ncH2 levels. However, polyadenylated ncH2 molecules accumulated upon PNPase silencing, suggesting that REXO2 and PNPase functionally cooperate in ncH2 removal. Such cooperation would align with the fact that REXO2 degrades nonadenylated or partially adenylated ncH2, whereas PNPase digests the polyadenylated pool. These cooperative functions of REXO2 and PNPase were also supported by our *in vitro* results, in which poly(A) tracts were not the preferred substrates of REXO2. The combined actions of REXO2 and PNPase ensure that ncH2 is kept at very low levels under normal conditions. Whether this transcript has any function remains to be studied, but our data imply that ncH2 molecules and other short RNAs that are not removed by REXO2 interfere with the function of the mitochondrial degradosome, a major RNA-degradation factor that controls the levels of mt-dsRNA and mitochondrial antisense transcripts (9,10,18,35). Consequently, the steady-state levels of these mtRNA species increase upon REXO2 inactivation, despite the fact that they are not direct REXO2 substrates. These *in cellulo* observations were supported by *in vitro* experiments that showed that nanoRNA removal by REXO2 increased the efficiency of tRNA-like degradation by the PNPase-SUV3 complex.

An alternative explanation for the increase in the steady-state levels of ncH2, antisense RNA, and dsRNA in REXO2-depleted mitochondria could be the upregulation of mtDNA transcription. Such a possibility is suggested by data from prokaryotes. The depletion of oligoribonuclease in *Pseudomonas aeruginosa* resulted in the accumulation of nanoRNAs, which are utilized as primers for transcription initiation and cause global alterations of gene expression (36). In line with this possibility, Nicholls *et al.* recently reported that nanoRNAs stimulate mtDNA transcription *in vitro* (34). The authors also observed a slight increase in mitochondrial transcription in the REXO2 knockdown mouse heart (34). In our experimental models, we did not find evidence that the upregulation of mt-dsRNA and the newly identified ncH2 transcript was attributable to an increase in mtDNA transcription. First, we analyzed the stability of mt-dsRNA and found, in line with our hypothesis, that REXO2 depletion resulted in the stabilization of mt-dsRNA. Second, we examined steady-state levels of ncH2 in REXO2-depleted cells in which transcription was inhibited and found no significant decrease in ncH2 levels. Third, we analyzed the mitochondrial transcription rate and found no upregulation of mtDNA transcription upon REXO2 silencing. In fact, a reduction of mtDNA transcription was observed in cells with depletion of the mitochondrial fraction of REXO2. These results do not necessarily contradict the findings of Nicholls *et al.* The different results may instead be attributable to the fact that we used actively proliferating cells that are likely more sensitive to disturbances in mtDNA expression. In fact, to explain why they observed only a slight effect of REXO2 knock-down on mtDNA transcription *in vivo*, Nicholls *et al.* suggested that highly proliferative cells are more affected by the loss of REXO2, because the clearance of nanoRNAs would be more important at increased proliferation rates. Therefore, the effects of REXO2 dysfunction may likely vary between models. Altogether, our findings indicate that the increase in steady-state levels of ncH2, antisense mtRNAs, and mt-dsRNA resulted from the absence of their degradation and not from an increase in mtDNA transcription. This does not necessarily contradict the work of others but rather highlights the importance of the clearance of short RNAs that otherwise directly or indirectly interfere with various mtRNA transactions, such as mtRNA degradation (present study) and mtDNA transcription (34). Thus, our data support the supposition of Nicholls *et al.* (34) that the dysfunction of REXO2, depending on the biological context, can have different consequences and manifestations.

Our *in vitro* experiments generated novel data on REXO2 substrate specificity. While the inability of the enzyme to degrade structured or double-stranded RNA was expected when considering the structural analysis of REXO2 and its prokaryotic homologs, we found that REXO2 degraded much longer single-stranded substrates than previously reported. In the case of some longer substrates, such as poly(U) homopolymers, degradation was very robust despite its distributive manner. Moreover, we found that adenine tracks were the least preferred substrate of REXO2, indicating that the enzyme possesses sequence specificity. We also found that the third nucleotide from the 3′ end of RNA determines the degradation efficiency of REXO2, in which adenine is the least optimal nucleotide. These findings contrast with an earlier report that suggested no apparent sequence specificity of REXO2 (28). This difference is explained by the choice of substrates that were used in our study and by others for the RNA decay assays. We used substrates of various lengths and sequences to study RNA degradation properties of REXO2. Other studies limited their experiments to only adenine homopolymers (28), which, according to our results, precluded the possibility of observing REXO2 sequence preference.

Nicholls *et al.* proposed that human REXO2 is a dedicated dinucleotidase (34). They observed the absence of the sequence specificity of REXO2 for diribonucleotide substrates, which is consistent with our data that indicated that REXO2 ‘senses’ the third nucleotide from the 3′ end. Thus, no sequence specificity should be expected for 2-nt-long substrates. Additionally, strong preference for dinucleotides was reported, which may imply a potential discrepancy with our findings that REXO2 degraded RNAs that were 10 nt long or longer (e.g. ncH2). These dissimilarities can be explained by the different experimental conditions, particularly the concentration of REXO2 protein and type of substrates that were used in the *in vitro* RNA degradation assays. Nicholls *et al.* performed the majority of their RNA degradation assays with REXO2 at concentrations of 0.25–4 nM (34), which is more than 10-times lower than the 50 nM concentration that was used by us. Importantly, when Nicholls *et al.* applied higher concentrations of REXO2 (32 and 64 nM) they did observe the full degradation of >2 nt RNA substrates (34), which is in agreement with our data. Unfortunately, they did not tested substrates that were longer than 5 nt, thus precluding further comparisons with the present results. Nevertheless, it should be noted that our results also indicated the preference of REXO2 for short substrates, particularly dinucleotides, as 2-nt degradation by-products were undetectable in our experiments. In summary, our data and Nicholls *et al.* (34) indicate that REXO2 degrades unstructured RNAs, with some preference for 2-nt substrates. However, our experiments revealed that the enzyme has sequence preference and is able to efficiently degrade even 25-nt-long substrates, depending on the sequence of the substrate and concentration of the enzyme. The exact concentrations of REXO2 in organelles are unknown, but pan-cellular analyses of HeLa cells by quantitative proteomics suggest ∼450 nM (37) or ∼1000 nM (38) concentrations of REXO2.

Our structural studies explain why REXO2 is unable to degrade structured or double-stranded RNA and why the enzyme exhibits a distributive mode of action. This results from the fact that the catalytic center of REXO2 can accommodate only single-stranded nucleic acids without an ability to continuously move along the substrate. Moreover, our analysis of REXO2 variants that harbor point mutations defines residues that are required for catalytic activity, highlighting the role of dimerization in formation of the active molecule, which is in agreement with a previous study (34). However, as we managed to determine the structure of REXO2 only with one substrate this data was not sufficient to decipher the structural basis of REXO2 sequence preference. Importantly, while our study was underway, other structures of REXO2 with RNA were published. These include REXO2 in complex with U_12_ (28) and REXO2 in complex with dinucleotides AA, AG and GG (39). Comparisons of our structure with the structures that were published by Kim *et al.* (39) shed light on the sequence preference of REXO2. Based on the structures of REXO2 in complex with dinucleotides (PDB ID: 6N6J and 6N6I) (39), the adenosine residue at the second position from the 3′ terminus is present in both anti and syn positions with a preference for the much less favorable syn conformation (Supplementary Figure S9). This is in contrast to other bases that occur only in anti conformation (Supplementary Figure S9). Moreover, our structure indicates that the third nucleotide from the 3′ end is exposed to the solvent and does not form any interactions with the protein. Therefore, slower RNA degradation by REXO2, which we observed when adenine was in the third position from the 3′ end, was likely attributable to the fact that the next cycle of hydrolysis requires transition of the adenine from the solvent to an unfavorable position.

Altogether, our work revealed REXO2 as an mtRNA surveillance factor that is indispensable for proper mtRNA metabolism. Our studies reinforce the concept that the effective regulations of RNA levels by degradation requires the combined action of ribonucleases with different specificities. In this respect, REXO2 is essential for removing short RNAs that otherwise could affect the activity of other RNA processing factors, e.g. the mitochondrial degradosome. We unveiled a peculiar sequence preference of REXO2. This 3′-5′ exoribonuclease pauses when an adenine residue occupies the third position from the 3′ end of the substrate. We identified a distinct mitochondrial transcript, ncH2, the levels of which are directly controlled by REXO2 RNA degradation activity. Functionally, our work and the recent study by Nicholls *et al.* (34) showed that REXO2 deficiency affects various aspects of mtRNA metabolism and results in different consequences, depending on the cellular context.

## DATA AVAILABILITY

The RNA-seq data that were generated in the present study are available in the GEO repository GSE137225, [https://www.ncbi.nlm.nih.gov/geo/query/acc.cgi?acc=GSE137225]. Atomic coordinates and structure factors for the reported crystal structures have been deposited in PDB (ID no. 6STY). Other relevant data are included in the paper and accompanying Supplementary Information.

## Supplementary Material

gkaa302_Supplemental_File

## Notes

Present address: Maciej Szewczyk, Department of Vertebrate Ecology and Zoology, Faculty of Biology, University of Gdańsk, Gdańsk 80-308, Poland.
