# Supplementary material for: Human REXO2 controls short mitochondrial RNAs generated by mtRNA processing and decay machinery to prevent accumulation of double-stranded RNA
Source: Nucleic Acids Res. 2020 May 4;48(10):5572–90. doi: 10.1093/nar/gkaa302 (PMC7261184; doi:10.1093/nar/gkaa302)
Supplement: gkaa302_Supplemental_File [file gkaa302_supplemental_file.pdf]

SUPPLEMENTARY FIGURES AND TABLES

Human REXO2 controls short mitochondrial RNAs generated by mtRNA processing and decay machinery to prevent accumulation of double-stranded RNA

Maciej Szewczyk[1,2,#], Deepshikha Malik[3], Lukasz S. Borowski[1,2], Sylwia D. Czarnomska[1,], Anna V. Kotrys[1], Kamila Klosowska-Kosicka[1], Marcin Nowotny[3] and Roman J. Szczesny[1]*

1. Institute of Biochemistry and Biophysics Polish Academy of Sciences, Warsaw, 02-106, Poland

2. Faculty of Biology, Institute of Genetics and Biotechnology, University of Warsaw, Warsaw, 02-106, Poland

3. Laboratory of Protein Structure, International Institute of Molecular and Cell Biology, Warsaw, 02-109, Poland

** – To whom correspondence should be addressed:*

Tel: +48 22 592 30 23; Email: rszczesny@ibb.waw.pl (R.J.S)

# – Current address: Department of Vertebrate Ecology and Zoology, Faculty of Biology, University of Gdańsk, Gdańsk, 80-308, Poland

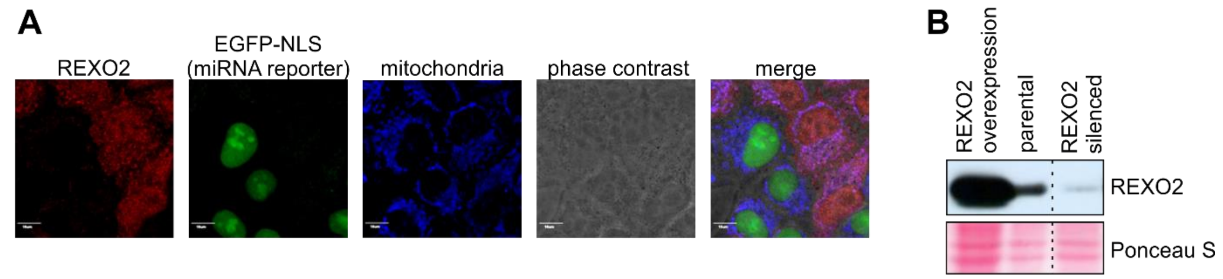

Supplementary Figure S1. Validation of anti-REXO2 antibodies specificity. (A) Immunofluorescence analysis of REXO2 level in REXO2-depleted cells (EGFP-NLS reporter) and parental HeLa cells (no reporter). Primary anti-REXO2 antibodies were visualized with anti-rabbit AlexaFluor555-labeled secondary antibody. Nuclei and mitochondria were stained with Hoechst and MitoTracker DeepRed, respectively. **(B)** Western blot analysis of REXO2 level in cells overexpressing REXO2 (HeLa cells transiently transfected with plasmid encoding REXO2_WT), parental HeLa cells or REXO2-depleted cells. PonceauS staining was used as a loading control.

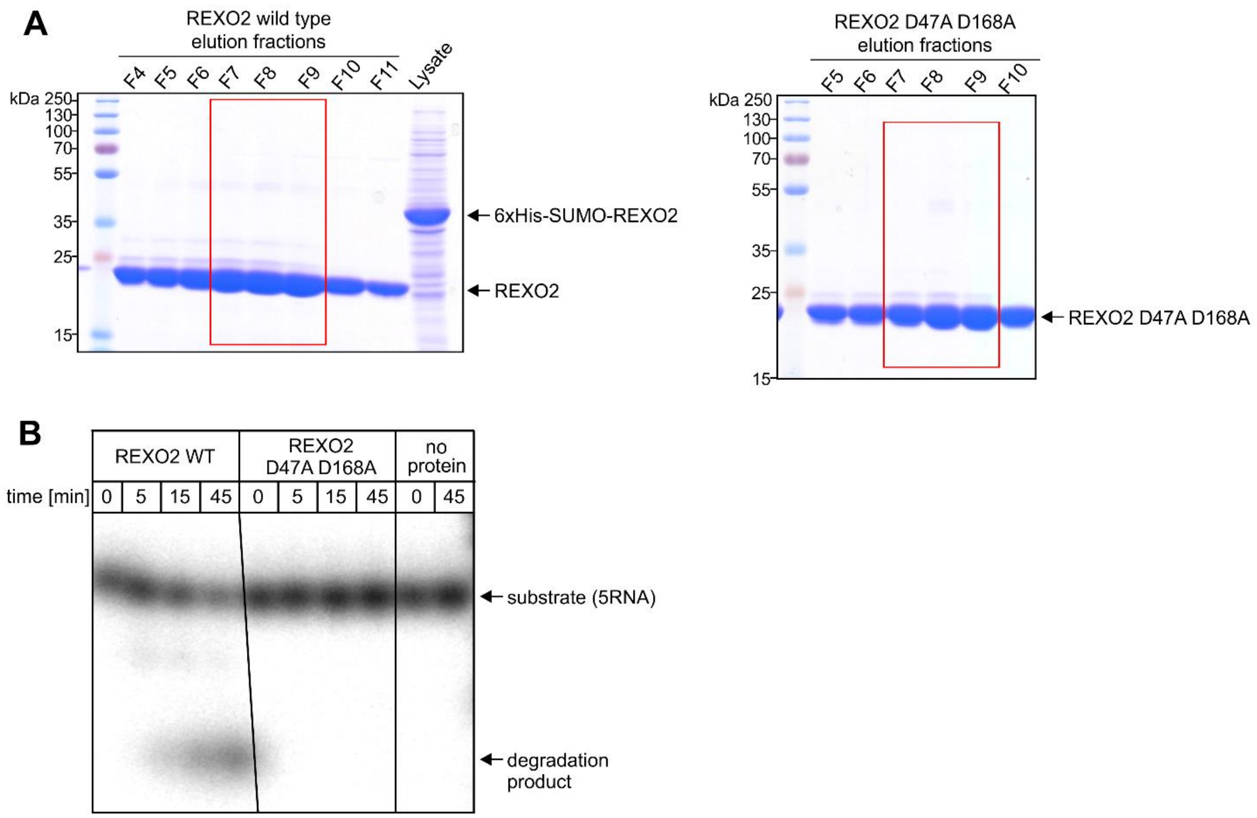

Supplementary Figure S2. Purification of recombinant REXO2. (A) SDS-PAGE analysis of recombinant REXO2 preparations. Red rectangles indicate fractions that were pooled and used in subsequent biochemical assays. **(B)** Results of *in vitro* degradation assay of radiolabeled 5-nt long RNA substrate by indicated recombinant REXO2 variants.

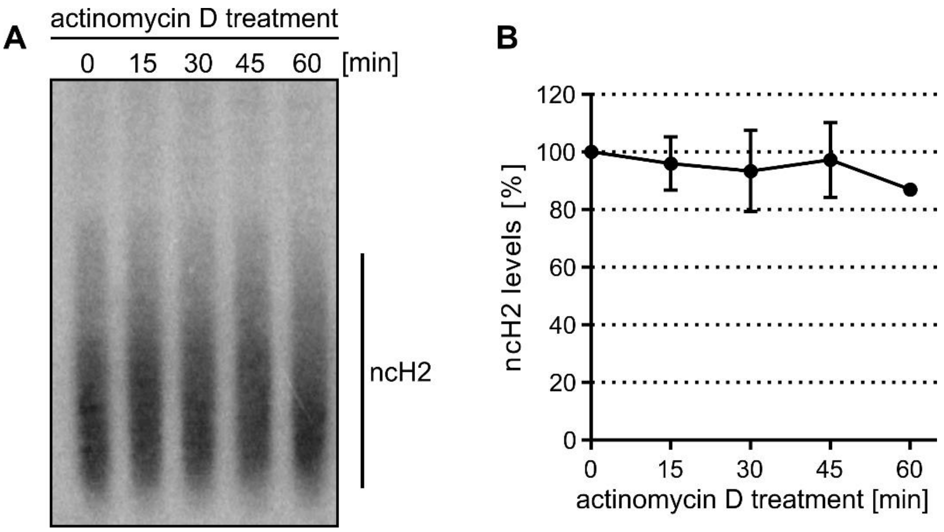

Supplementary Figure S3. Quantification of ncH2 transcript in REXO2-silenced HeLa cells treated with transcription inhibitor. REXO2-depleted HeLa cells were treated (or not) with actinomycin D) for the indicated time and then the level of ncH2 was measured with northern blot analysis. Level of ncH2 in untreated cells (point 0) was set as 100%. Mean values are shown. Error bars represent SEM. (**A**) Results of northern blot hybridization. (**B**) Quantification of two independent experiments. Mean values are shown. Error bars represent SEM.

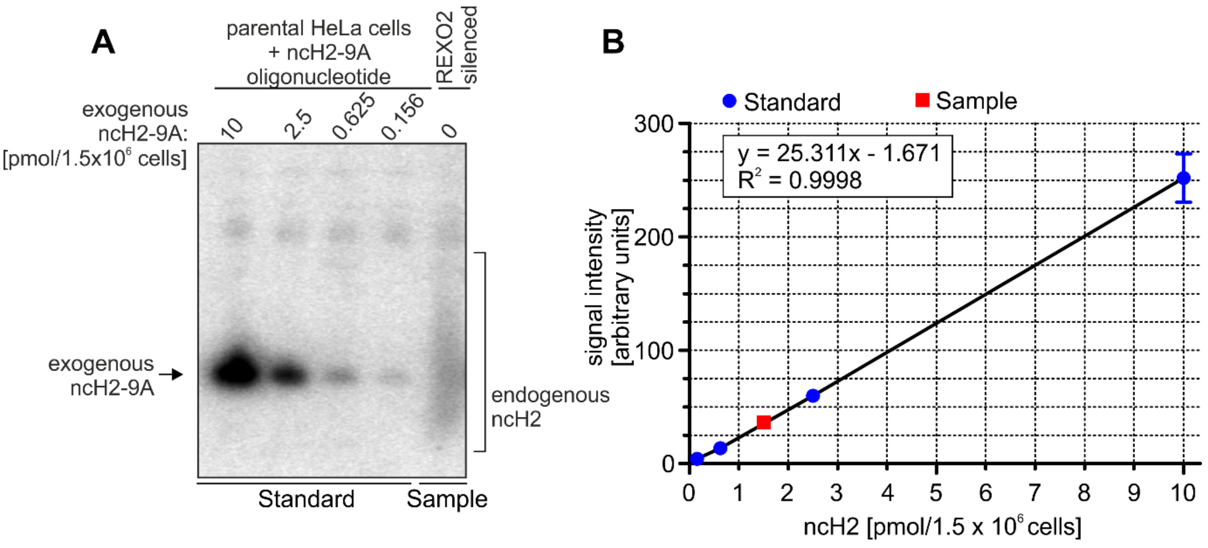

Supplementary Figure S4. Quantification of ncH2 transcript in HeLa cells. (A) Quantitative northern blot analysis of ncH2 level. The same number (1.5 mln) of parental or REXO2-depleted HeLa cells was collected and indicated quantities of synthetic ncH2-9A RNA oligonucleotide were added to parental HeLa pellets prior to RNA isolation. 2 µg of total RNA were loaded per lane. Further steps were performed as described in the main text. **(B)** Calculation of ncH2 level in REXO2-depleted cells using data from quantitative northern blot (A). The calculated ncH2 quantity equaled 1.504 pmol per 1.5 mln cells, corresponding to around 604,000 ncH2 copies per cell. Graph was based on the results of three independent experiments. Mean values are shown. Error bars represent SEM.

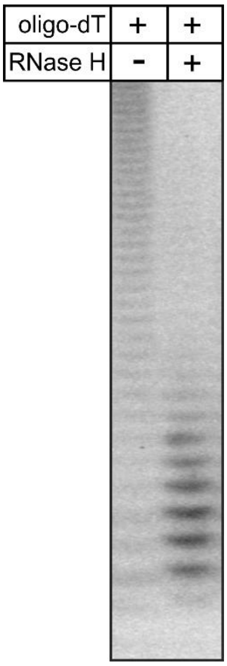

Supplementary Figure S5. ncH2 transcript is polyadenylated. RNA isolated from PNPase-depleted cells was annealed to $(dT)_{18}$ oligonucleotide and subsequently treated (or not) with RNase H. Reaction products were analyzed by high-resolution northern blot with ncH2 detecting oligoprobe as described in the main text.

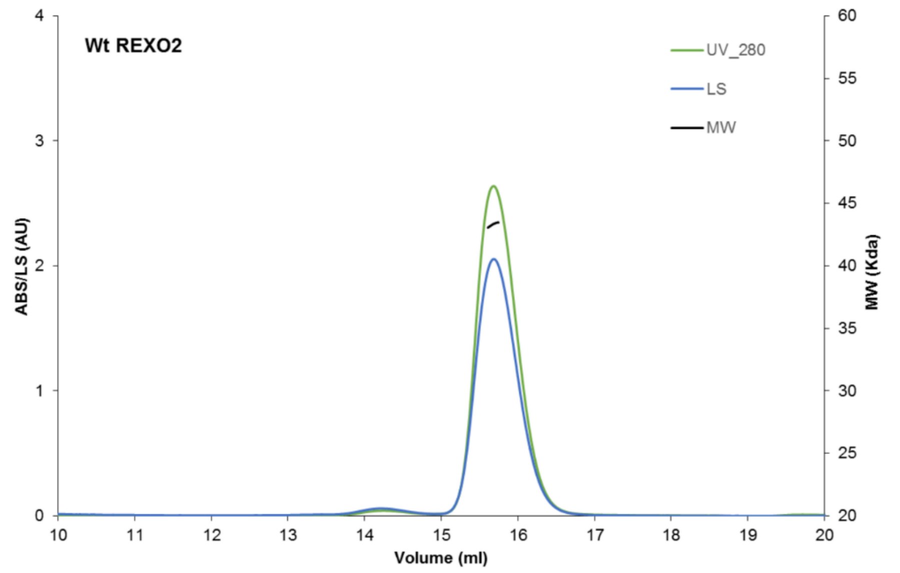

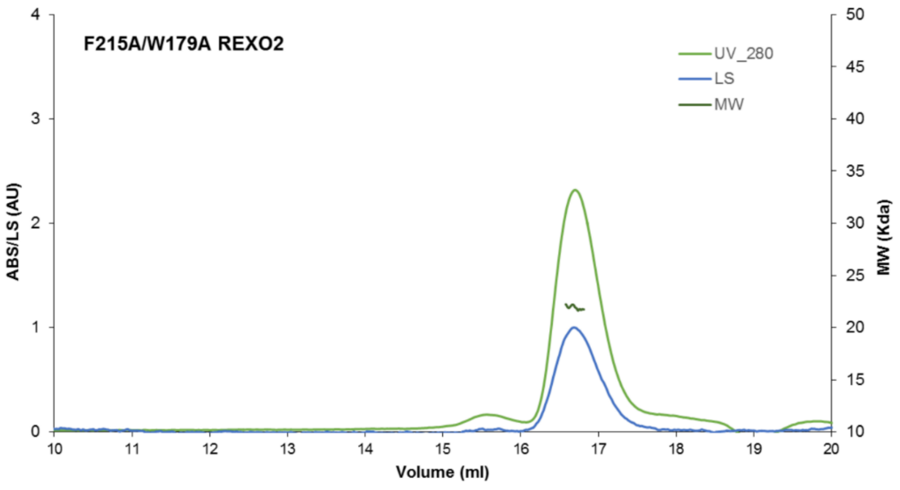

Protein	Theoretical Mass of monomer (KDa)	MALS Calculated Mass (KDa)
WT REXO2	24	44.48
F215/W179A REXO2	24.1	21.75

Supplementary Figure S6. REXO2 dimerization. Elution profile of REXO2 from a gel filtration column (Superdex 200 Increase 10/300, GE Healthcare). Absorbance at 280 nm is shown in green and light scattering signal in blue (left axis). The molecular weight calculated based on multi-angle light scattering (MALS) is shown in black (right axis). The upper panel shows elution profile of the wild type protein and the lower one of F215A/W179A variant. Theoretical as well as MALS-measured mass are presented in the table.

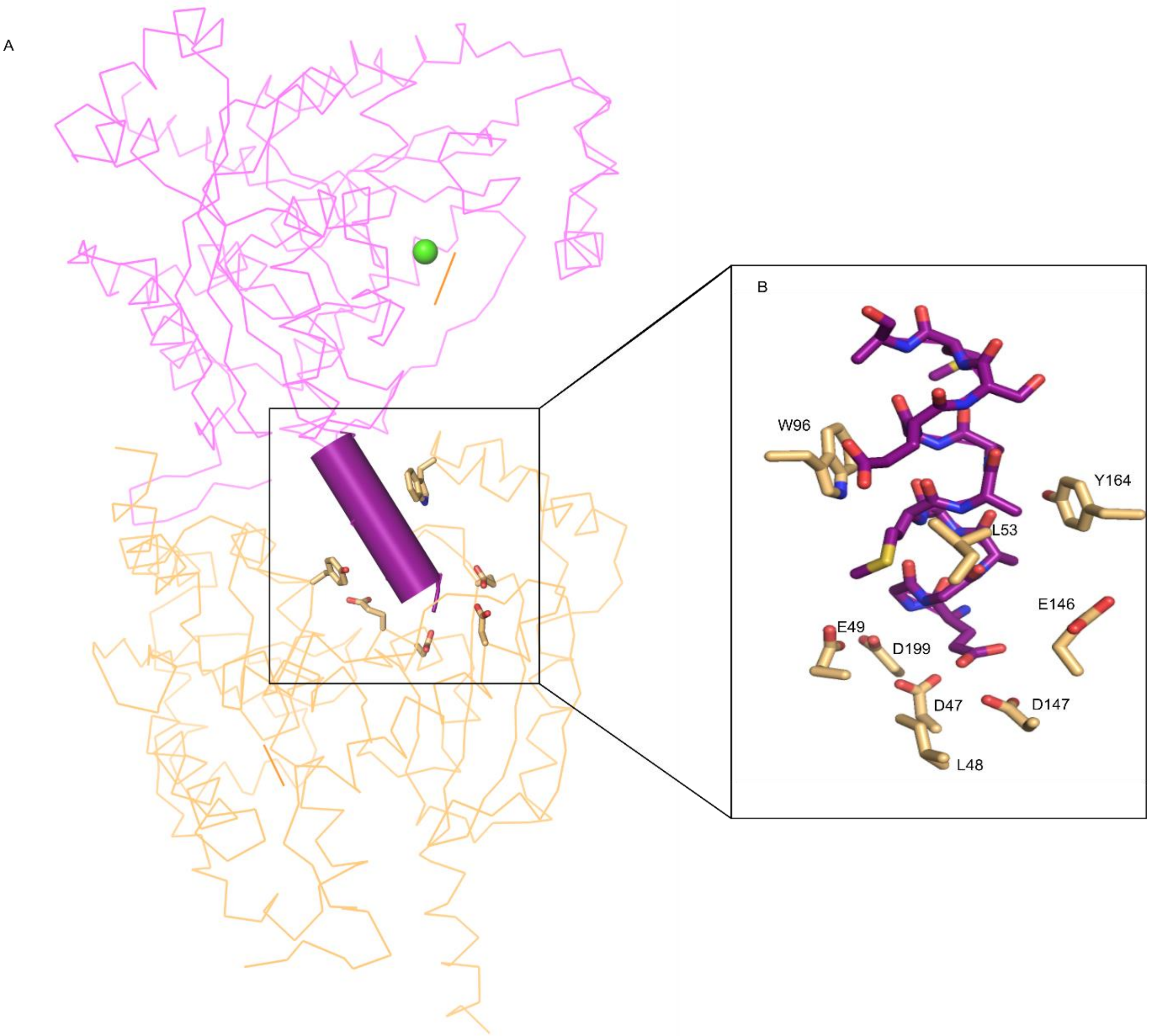

Supplementary Figure S7. N-terminal helix of REXO2. **(A)** Crystal contact observed in REXO2 structure. Interacting REXO2 dimers are shown as orange and purple wire with N-terminal helix (residues 28-37) of one protomer of the latter dimer is shown as a cylinder. The residues interacting with the helix are shown as sticks. **(B)** Close-up view of the interaction.

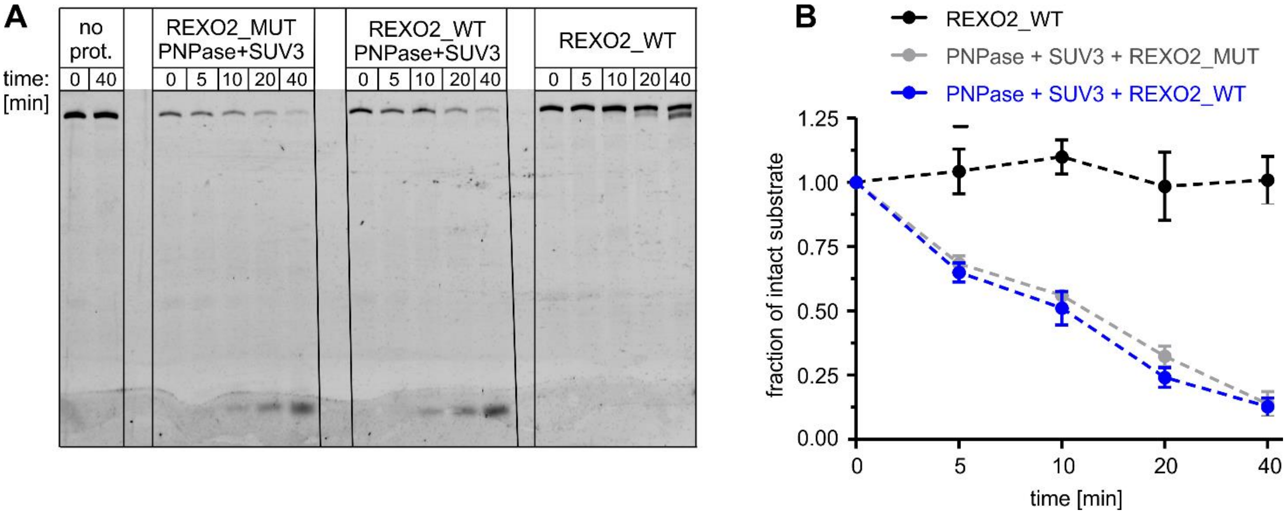

Supplementary Figure S8. REXO2 has no effect on tRNA-like degradation by the degradosome in the absence of an excess of nanoRNA. Figure related to Figure 7 from the main text. (**A**) In vitro degradation of tRNA-like oligonucleotide by recombinant REXO2, mitochondrial degradosome (SUV3 and PNPase), and their combined action. REXO2_WT, wild-type REXO2; mut, catalytically inactive REXO2 (D47A D168A); no prot., no protein control. Reactions were performed in the absence of an excess of nanoRNA (5RNA). (**B**) Quantification of tRNA-like decay that is shown in A. The data are expressed as the mean ±SEM of four independent experiments.

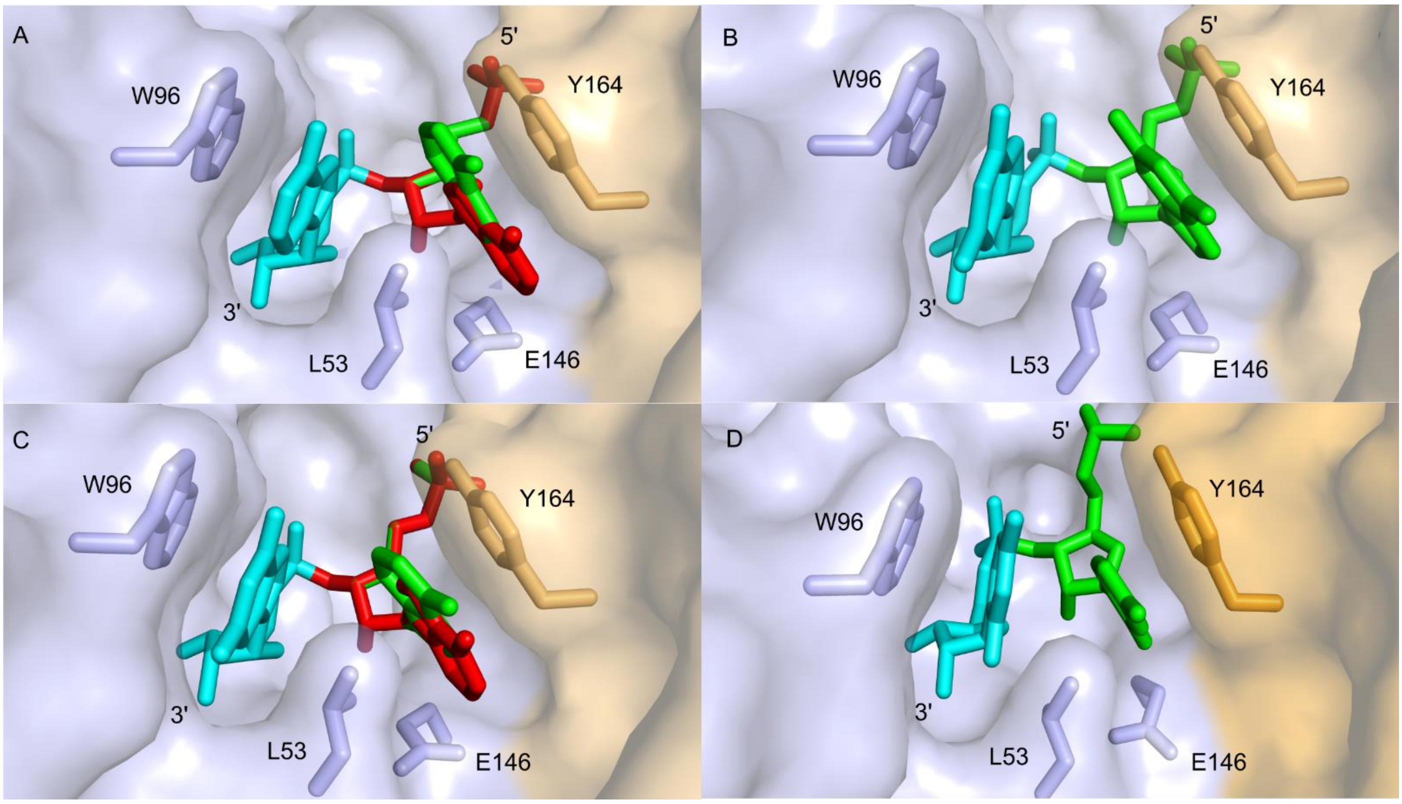

Supplementary Figure S9. Surface representation of aromatic clamp between W96 and Y164 with substrates of different sequences. In each panel protomer A is light orange and protomer B is light blue. The nucleotide at the active site is colored in cyan and the nucleotide at second position from the active site is colored in green or red depending on its conformation (anti or syn, respectively). **(A)** Aromatic clamp with AA as the substrate (pdb id 6n6j). The adenosine base present at the second position from 3′ end exists in two conformations – syn (red) and anti (green). **(B)** Aromatic clamp with GG as the substrate (pdb id: 6n6i). The guanosine base at the second position present only in anti conformation. **(C)** Aromatic clamp with AG as the substrate (pdb id: 6n6k). The adenosine base at the 5′ end is present in syn (red) and anti (green) conformation. **(D)** Aromatic clamp with UC as the substrate (present work). The uracil base at the penultimate position from the active site present only in anti conformation.

SUPPLEMENTARY TABLES

Table S1. Generated DNA constructs. Vectors pKK as described by Szczesny *et al.*

ID	Encoded feature	Vector	Functionality	Application
pRS1038	REXO2 miR	pKK-RNAi-nucEGFP-miR-TEV-FLAG	Endogenous REXO2 silencing	Generation of stable HeLa cell line
pRS1039	REXO2 miR	pKK-RNAi-nucCHERRY-miR-TEV-FLAG	Endogenous REXO2 silencing	Generation of stable HeLa cell line
pRS1040	REXO2 miR, REXO2	pKK-RNAi-nucEGFP-miR-TEV-FLAG	Endogenous REXO2 silencing and concomitant expression of miR insensitive REXO2_WT	Generation of stable HeLa cell line
pRS1041	REXO2 miR, REXO2 D168A	pKK-RNAi-nucCHERRY-miR-TEV-FLAG	Endogenous REXO2 silencing and concomitant expression of miR insensitive REXO2_MUT	Generation of stable HeLa cell line
pRS1042	REXO2 miR, REXO2 M33A, K222A	pKK-RNAi-nucCHERRY-miR-TEV-FLAG	Endogenous REXO2 silencing and concomitant expression of miR insensitive REXO2_mit	Generation of stable HeLa cell line
pRS1043	REXO2 miR, REXO2 M33A, K222A, D168A	pKK-RNAi-nucCHERRY-miR-TEV-FLAG	Endogenous REXO2 silencing and concomitant expression of miR insensitive REXO2_mit_MUT	Generation of stable HeLa cell line
pRS1044	REXO2 miR, Δ32_REXO2	pKK-RNAi-nucCHERRY-miR-TEV-FLAG	Endogenous REXO2 silencing and concomitant expression of miR insensitive REXO2_nonmit	Generation of stable HeLa cell line
pRS1103	REXO2 miR, Δ32_REXO2 D168A	pKK-RNAi-nucCHERRY-miR-TEV-FLAG	Endogenous REXO2 silencing and concomitant expression of miR insensitive REXO2_nonmit_MUT	Generation of stable HeLa cell line
pRS673	6xHis-SUMO-TEV-Δ28_REXO2	pET28	Mature 6xHis-SUMO-TEV-REXO2_WT fusion	Purification of indicated REXO2
pRS675	6xHis-SUMO-TEV-Δ28_REXO2 D47A D168A	pET28	Mature 6xHis-SUMO-TEV-REXO2_MUT fusion	Purification of indicated REXO2
pRS740	6xHis-SUMO-TEV-Δ28_REXO2 ΔN37	pET28	Mature 6xHis-SUMO-TEV-REXO2_MUT fusion	Purification of indicated REXO2
pRS741	6xHis-SUMO-TEV-Δ28_REXO2 L53A	pET28	Mature 6xHis-SUMO-TEV-REXO2_MUT fusion	Purification of indicated REXO2
pRS742	6xHis-SUMO-TEV-Δ28_REXO2 W96A	pET28	Mature 6xHis-SUMO-TEV-REXO2_MUT fusion	Purification of indicated REXO2
pRS743	6xHis-SUMO-TEV-Δ28_REXO2 E146A	pET28	Mature 6xHis-SUMO-TEV-REXO2_MUT fusion	Purification of indicated REXO2
pRS744	6xHis-SUMO-TEV-Δ28_REXO2 Y164A	pET28	Mature 6xHis-SUMO-TEV-REXO2_MUT fusion	Purification of indicated REXO2
pRS745	6xHis-SUMO-TEV-Δ28_REXO2 D47/E49A	pET28	Mature 6xHis-SUMO-TEV-REXO2_MUT fusion	Purification of indicated REXO2
pRS746	6xHis-SUMO-TEV-Δ28_REXO2 E49A	pET28	Mature 6xHis-SUMO-TEV-REXO2_MUT fusion	Purification of indicated REXO2
pRS747	6xHis-SUMO-TEV-Δ28_REXO2 D147A	pET28	Mature 6xHis-SUMO-TEV-REXO2_MUT fusion	Purification of indicated REXO2
pRS748	6xHis-SUMO-TEV-Δ28_REXO2 D199A	pET28	Mature 6xHis-SUMO-TEV-REXO2_MUT fusion	Purification of indicated REXO2
pRS749	6xHis-SUMO-TEV-Δ28_REXO2 F215/W179A	pET28	Mature 6xHis-SUMO-TEV-REXO2_MUT fusion	Purification of indicated REXO2

Table S2. Primers used in the study.

ID	Gene	Sequence	Application
RSZ270	7S RNA	TAATACGACTCACTATAGGGGAATGTCTGCACAGCCAC	Northern blot (strand-specific, riboprobe)
RSZ271	7S RNA	ATTTAGGTGACACTATAGAATTGAAATCTGGTTAGGCTGG	Northern blot (strand-specific, riboprobe)
RSZ124	MT-RNR1	ATTACACATGCAAGCATCCC	Northern blot
RSZ125	MT-RNR1	CACGAAATTGACCAACCCTG	Northern blot
RSZ290	MT-RNR2	TAATACGACTCACTATAGGGCACACCCAATTGGACCAATC	Northern blot (strand-specific, riboprobe)
RSZ291	MT-RNR2	ATTTAGGTGACACTATAGAAAGACAGCTGAACCCTCGTG	Northern blot (strand-specific, riboprobe)
RSZ655	tRNA-like	ACGTTCTCCTGATCAAATATCACTCTCCTAC	Northern blot (oligoprobe)
RSZ943	oriL primer (mtDNA 5747-80)	CTTCAAACCTGCCGGGGCTTCTCCCGCCTTTTTT	Northern blot (oligoprobe)
RSZ944	nc-OL (mtDNA 5730-46)	CCCGGCGGCGGGAGAAG	Northern blot (oligoprobe)
RSZ946	ncH2 (mtDNA 561-76)	GGGGGTGTCTTT	Northern blot (oligoprobe)
RSZ312	$(dT)_{16}$	TTTTTTTTTTTTTTTT	RNase H treatment
RSZ638	REXO2	GAAGGTGGCGCAGCCGCGGCGGCAGGGGAGAG	M33A mutagenesis
RSZ639	REXO2	CTCTCCCCTGCCGCCGCGGCTGCGCCACCTTC	M33A mutagenesis
RSZ646	REXO2	GATGCGAACGCGAGGAACATTATAGAAAATGGG	K222A mutagenesis
RSZ647	REXO2	CCCATTTTCTATAATGTTCCTCGCGTTCGCATC	K222A mutagenesis
RSZ629	REXO2	TATAGAATAATTGCTGTGAGCACTGTTAAG	D168A mutagenesis
RSZ630	REXO2	CTTAACAGTGCTCACAGCAATTATTCTATA	D168A mutagenesis
RSZ636	REXO2	GATGGTCTGGGTGGCCCTGGCGATGACAGGATTGG	D47A mutagenesis
RSZ637	REXO2	CCAATCCTGTCATCGCCAGGGCCACCCAGACCATC	D47A mutagenesis
NA	REXO2	CTGAGCCATGCTCTCCATGGATCCACCGGT	ΔN37 mutagenesis
NA	REXO2	ACCGGTGGATCCATGGAGAGCATGGCTCAG	ΔN37 mutagenesis
NA	REXO2	CAAATTCATATTCTTCTGGATACGCGCGTCTGCACAGTTCTTTAACAG	W179A mutagenesis
NA	REXO2	CTGTTAAAGAACTGTGCAGACGCGCGTATCCAGAAGAATATGAATTTG	W179A mutagenesis
NA	REXO2	CTCTTCTTTTCATCTATTTTTTTCTTGGCGATGTTATTTCGGTAAAAC TGAAGCTC	F215A mutagenesis
NA	REXO2	GAGCTTCAGTTTTACCGAAATAACATCGCCAAGAAAAAAATAGATGAA AAGAAGAG	F215A mutagenesis
NA	REXO2	GGTCCTTCTCAATGTCTAATCCTGTCATCTCCAGGTCC	L53A mutagenesis
NA	REXO2	GGACCTGGAGATGACAGGAGCGGACATTGAGAAGGACC	L53A mutagenesis
NA	REXO2	ATGCTCCTTACACGCATCTGACATGCTGTCCAGCAACTC	W96A mutagenesis
NA	REXO2	GAGTTGCTGGACAGCATGTCAGATGCGTGTAAGGAGCAT	W96A mutagenesis
NA	REXO2	ATTTGTCAAGAAACTTCTTATCTGCATGAACTGAATTTCCTGCAAGT	E146A mutagenesis
NA	REXO2	ACTTGCAGGAAATTCAGTTCATGCAGATAAGAAGTTTCTTGACAAAT	E146A mutagenesis
NA	REXO2	TGCTCACATCAATTATTCTAGCATGAAGATGTTTCATGAACTGGGGCATG	Y164A mutagenesis
NA	REXO2	CATGCCCCAGTTCATGAAACATCTTCATGCTAGAATAATTGATGTGAGCA	Y164A mutagenesis
NA	REXO2	TGTATTTGTCAAGAAACTTCTTAGCTTCATGAACTGAATTTCCTGCA	D147A mutagenesis
NA	REXO2	TGCAGGAAATTCAGTTCATGAAGCTAAGAAGTTTCTTGACAAATACA	D147A mutagenesis
NA	REXO2	CCAATCCTGTCATCGCCAGGTCCACCCAG	E49A mutagenesis
NA	REXO2	CTGGGTGGACCTGGCGATGACAGGATTGG	E49A mutagenesis
NA	REXO2	GATGCTTTCACTAATGTCAGCAAGTGCCCTATGAGAAGC	D199A mutagenesis
NA	REXO2	GCTTCTCATAGGGCACTTGCTGACATTAGTGAAAGCATC	D199A mutagenesis

Single underline – T7 RNA polymerase promoter sequence, Double underline – SP6 RNA polymerase promoter sequence, NA – not applicable

Table S3. Oligoribonucleotides used in the study. Synthetized by FutureSynthesis (Poznan, Poland)

ID	Name	Sequence<br>(modified nucleotide is double underlined)	Application
1	5RNA	CGACU	RNA degradation assay
6	10RNA	CGACUGGAGC	RNA degradation assay
10	22RNA	CGACUGGAGCACGAGGACACUG	RNA degradation assay
15	34RNA	CGACUGGAGCACGAGGACACUGACAUGGACUGAA	RNA degradation assay
16	44RNA	CGACUGGAGCACGAGGACACUGACAUGGACUGAAGGAGUAGAAA	RNA degradation assay
65	C80RNA	GCUGAUCAACCCUACAUGUGUAGGUAACCCUAACCCUAACCCUAAGGAC AACCCUAGUGAAGCUUGUAACCCUAGGAGCU	RNA degradation assay
67	tRNA-like	(fluorescein)CUGUGACUAGUAUGUUGAGUCCUGUAAGUAGGAGAG UGAUAUUUGAUCAGGAGAACGUGGUUACUAGCACAGAGAGUUCUCCA	RNA degradation assay
79	25A	(fluorescein)AAAAAAAAAAAAAAAAAAAAAAAAA	RNA degradation assay
80	25U	(fluorescein)UUUUUUUUUUUUUUUUUUUUUUUUU	RNA degradation assay
100	RNA10comp-5A	GCUCCAGUCGAAAAA	RNA degradation assay
114.1	RNA-17	CCCCACCACCAUCACUU	RNA degradation assay
116	ncH2	AAAGACACCCCCCACA	RNA degradation assay
117	RNA5_2	GAUCG	RNA degradation assay
118	nc-OL	CUUCUCCCGCCGCCGGG	RNA degradation assay
119	oriL primer	AAAAAAGGCGGGAGAAGCCCCGGCAGGUUUGAAG	RNA degradation assay
120	AAAAA-AGUCG	AAAAAAGUCG	RNA degradation assay
98	AGUCG-AAAAA	AGUCGAAAAA	RNA degradation assay
122	25-AAUU	(fluorescein)UUAUUAUUAUUAUUAUUAUAAUUAU	RNA degradation assay
123	ncH2-9A	AAAGACACCCCCCACAAAAAAAAAA	RNA degradation assay
NA	RNA_3	ACCGGGA	Co-crystalization
NA	RNA_36	ACCGGGC	Co-crystalization
NA	RNA_13	ACCGGGCG	Co-crystalization
NA	RNA_4	UCCUUAGGA	Co-crystalization
NA	RNA_8	UUCUUAGG	Co-crystalization
NA	RNA_5	CCCGGCUCAUC	Co-crystalization

NA – not applicable

Table S4. Data collection and refinement statistics. Statistics for the highest-resolution shell are shown in parentheses.

Data collection	*REXO2-RNA*
Space group	$P\ 3_2$
Cell dimensions	
a, *b*, *c* (Å)	88.3, 88.3, 123.8
α, β, γ (°)	90, 90, 120
Resolution (Å)	48.1 - 3.15 (3.34 - 3.15)
$CC_{1/2}$	99.4 (84.7)
$I / \sigma I$	14.82 (3.08)
Completeness (%)	99.4 (96.8)
Redundancy	5.1 (4.9)
**Refinement**	
Resolution (Å)	48.1 - 3.15
No. of unique reflections	18626 (1847)
R_{work} / R_{free} (%)	16.4/22.3
No. atoms	6026
Protein	5924
Nucleic acid	93
Ion/Water	9
B-factors ($Å^2$)	82.0
Protein	82.0
Nucleic acid	133.3
Ion/Water	73.7
RMSD	
Bond length (Å)	0.003
Bond angle (°)	0.66

SUPPLEMENTARY METHODS

RNase H treatment

10 µg RNA was mixed with 200 picomoles of $(dT)_{16}$ oligonucleotide and heat denatured for 10 minutes at 85 °C in 1x annealing buffer (25 mM Tris-HCl pH 7.5, 1 mM EDTA, 50 mM NaCl) in a final volume of 20 µl. Reaction mixture was then slowly cooled to room temperature, allowing annealing of $(dT)_{16}$ oligonucleotide with RNA poly(A) tails. Next, 5 µl of 10x RNase H buffer (200 mM Tris-HCl pH 7.8, 400 mM KCl, 80 mM MgCl2, 10 mM DTT), 0.5 µl of RiboLock™ Ribonuclease Inhibitor (Thermo Scientific) and 1 µl (2U) RNase H (Invitrogen) was added and the total reaction volume was brought to 50 µl with nuclease-free water, followed by 1 hour incubation at 37 °C. Reaction was stopped by the addition of 1 µl of 0.5 M EDTA. Next, RNA was purified by ethanol precipitation and resuspended in nuclease-free water. Finally, 3 µg of purified RNase H treated RNA was analyzed by high-resolution northern blot with ncH2 oligoprobe as described in the main text.

SEC-MALS analysis

WT and mutant proteins were analyzed on Superdex 200 Increase 10/300 column (GE Healthcare) at room temperature. The column was equilibrated with buffer containing 100 mM NaCl, 20 mM HEPES pH 7.5, 0.5 mM TCEP at 0.5ml/min. Three in-line detectors: UV absorbance, MALS (DAWN HELEOS-II, Wyatt Technology), and differential refractometer (Optiab T-rEX,Wyatt Technology) were used for analysis of

protein elution profile. ASTRA software (Wyatt Technology) was used for data processing and molecular weight calculation.

Analysis of ncH2 in actinomycin D treated cells

Cells expressing miRNA targeting REXO2 were cultured for 72h with tetracycline (100 ng/ml). 72h after induction, cells were treated with actinomycin D (Sigma, A1410) at a final concentration of 5 µg/ml. After incubation, the medium was removed and the cells were lysed in 1 ml TRI Reagent (Sigma). RNA was isolated according to the manufacturer's instructions and 10 µg of RNA was separated on a 15% denaturing polyacrylamide gel in TBE with 8M urea. A wet transfer was carried out in 0.5x TBE for 2h at a constant voltage of 60V on the Amersham Hybond-N+ membrane (GE Healthcare). RNA was cross-linked by UV. Then northern hybridization was carried out with an ncH2 specific probe. Radioactively labeled oligonucleotide RSZ946 GGGGGTGTCTTT was used as the probe. Hybridization was carried out overnight at 37 °C in PerfectHyb buffer (Sigma). The membrane was washed twice with 2x SSC at 37 °C for 10 min. Phosphoimaging was performed on a Typhoon FLA 9000 scanner (GE Healthcare). Data were analyzed using Multi Gauge V3.0 software (FujiFilm).

SUPPLEMENTARY REFERENCE

Szczesny RJ, Kowalska K, Klosowska-Kosicka K, Chlebowski A, Owczarek EP, Warkocki Z, Kulinski TM, Adamska D, Affek K, Jedroszkowiak A, Kotrys AV, Tomecki R, Krawczyk PS, Borowski LS, Dziembowski A. Versatile approach for functional analysis of human proteins and efficient stable cell line generation using FLP-mediated recombination system. PLoS One. 2018 Mar 28;13(3):e0194887. PubMed PMID: 29590189
